# Altered immune and metabolic molecular pathways drive islet cell dysfunction in human type 1 diabetes

**DOI:** 10.1172/JCI195267

**Published:** 2025-09-30

**Authors:** Theodore dos Santos, Xiao-Qing Dai, Robert C. Jones, Aliya F. Spigelman, Hannah M. Mummey, Jessica D. Ewald, Cara E. Ellis, James G. Lyon, Nancy Smith, Austin Bautista, Jocelyn E. Manning Fox, Norma F. Neff, Angela M. Detweiler, Michelle Tan, Rafael Arrojo e Drigo, Jianguo Xia, Joan Camunas-Soler, Kyle J. Gaulton, Stephen R. Quake, Patrick E. MacDonald

**Affiliations:** 1 Department of Pharmacology and; 2 Alberta Diabetes Institute, University of Alberta, Edmonton, Alberta, Canada.; 3 Department of Bioengineering, Stanford University, Stanford, California, USA.; 4 Chan Zuckerberg Biohub, San Francisco, California, USA.; 5 Department of Applied Physics, Stanford University, Stanford, California, USA.; 6 Bioinformatics and Systems Biology Program, University of California San Diego, La Jolla, California, USA.; 7 European Bioinformatics Institute (EMBL-EBI), European Molecular Biology Laboratory, Wellcome Genome Campus, Hinxton, United Kingdom.; 8 Department of Molecular Physiology and Biophysics and; 9 Center for Computational Systems Biology, Vanderbilt University, Nashville, Tennessee, USA.; 10 Department of Microbiology & Immunology, McGill University, Montreal, Quebec, Canada.; 11 Department of Medical Biochemistry and Cell Biology, Institute of Biomedicine and; 12 Wallenberg Centre for Molecular and Translational Medicine, Sahlgrenska Academy, University of Gothenburg, Sweden.; 13 Department of Pediatrics, Pediatric Diabetes Research Center, University of California San Diego, La Jolla, California, USA.; 14 Division of Metabolism, Endocrinology & Diabetes, University of Michigan, Ann Arbor, Michigan, USA.

**Keywords:** Autoimmunity, Cell biology, Endocrinology, Diabetes, Islet cells

## Abstract

Type 1 diabetes (T1D) is characterized by the autoimmune destruction of most insulin-producing β cells, along with dysregulated glucagon secretion from pancreatic α cells. We conducted an integrated analysis that combines electrophysiological and transcriptomic profiling, along with machine learning, of islet cells from T1D donors. The few surviving β cells exhibit altered electrophysiological properties and transcriptomic signatures indicative of increased antigen presentation, metabolic reprogramming, and impaired protein translation. In α cells, we observed hyperresponsiveness and increased exocytosis, which are associated with upregulated immune signaling, disrupted transcription factor localization, and lysosome homeostasis, as well as dysregulation of mTORC1 complex signaling. Notably, key genetic risk signals for T1D were enriched in transcripts related to α cell dysfunction, including MHC class I, which were closely linked with α cell dysfunction. Our data provide what we believe are novel insights into the molecular underpinnings of islet cell dysfunction in T1D, highlighting pathways that may be leveraged to preserve residual β cell function and modulate α cell activity. These findings underscore the complex interplay between immune signaling, metabolic stress, and cellular identity in shaping islet cell phenotypes in T1D.

## Abstract 2

We analyzed pancreatic islet cells from donors with type 1 diabetes to define pathways and mechanisms responsible for altered insulin and glucagon in this disease.

## Graphical abstract

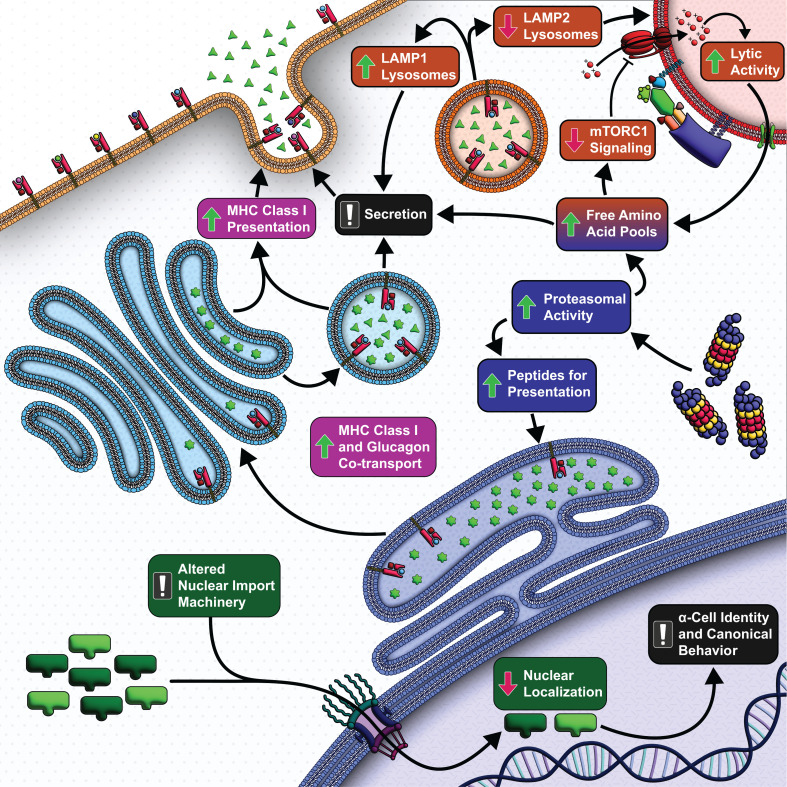

## Introduction

Type 1 diabetes (T1D) is defined by a near-complete loss of circulating insulin and dysregulated glucagon secretion. However, the autoimmune destruction of insulin-producing β cells in T1D is incomplete, as circulating insulin or C-peptide (1) and insulin-positive pancreatic cells are still detectable after many years (2). Indeed, circulating C-peptide levels in T1D correlate with lower microvascular complications (3). Even before T1D onset, β cells may be dysfunctional (4–6), although it is debated whether surviving β cells in established disease are functionally normal (7, 8) or abnormal (9–14). Molecular profiling suggests increased inflammatory and ER stress signatures in T1D β cells (9, 15–18).

Circulating glucagon is elevated at normoglycemia in T1D and not appropriately stimulated upon hypoglycemia (19). Individuals with T1D show elevated glucagon responses in mixed meal tolerance tests (20) and increased glucagon sensitivity to glucose-dependent insulinotropic polypeptide (GIP) (21). In vitro, glucagon secretion from islets of donors with T1D is dysregulated (7) and also appears elevated from islets of autoantibody-positive donors in response to cAMP-raising stimuli (22). Molecular profiling reveals increased MHC class I (MHC-I) on T1D α cells (23, 24), consistent with increased inflammatory signaling and ER stress (7, 24) and changes in expression of α cell identity markers (16, 25).

Here, we investigate mechanisms of islet cell dysfunction through linked electrophysiological and transcriptomic analyses. Islets from T1D donors, most with long-standing disease, had minimal insulin but retained some β cells with altered electrophysiological phenotypes, indicating dysfunction. Although there were few β cells, transcriptomic changes suggested increased antigen presentation, a shift toward glycolysis, and downregulation of translation, indicating metabolic reprogramming linked to autoimmune activity. T1D α cells showed hyperresponsiveness and increased activity. Molecular profiling indicated disrupted α cell identity and regulatory control, alterations to MHC-I and glucagon compartmentalization, and mammalian/mechanistic target of rapamycin complex 1 (mTORC1) dysregulation linked to excitability and exocytosis. Additionally, alterations in lysosomal balance and upregulated proteasomes indicate chronic cellular stress affecting protein turnover. These mechanisms contribute to the hyperactive, glucose-unresponsive phenotype in T1D α cells. Notably, genetic risk alleles for T1D were enriched in transcripts associated with α cell dysfunction. Our findings highlight distinct dysfunctional states in T1D α and β cells driven by transcriptomic and electrophysiological reprogramming.

## Results

### Islet isolation and cell phenotyping from donors with T1D.

**Figure 1:**
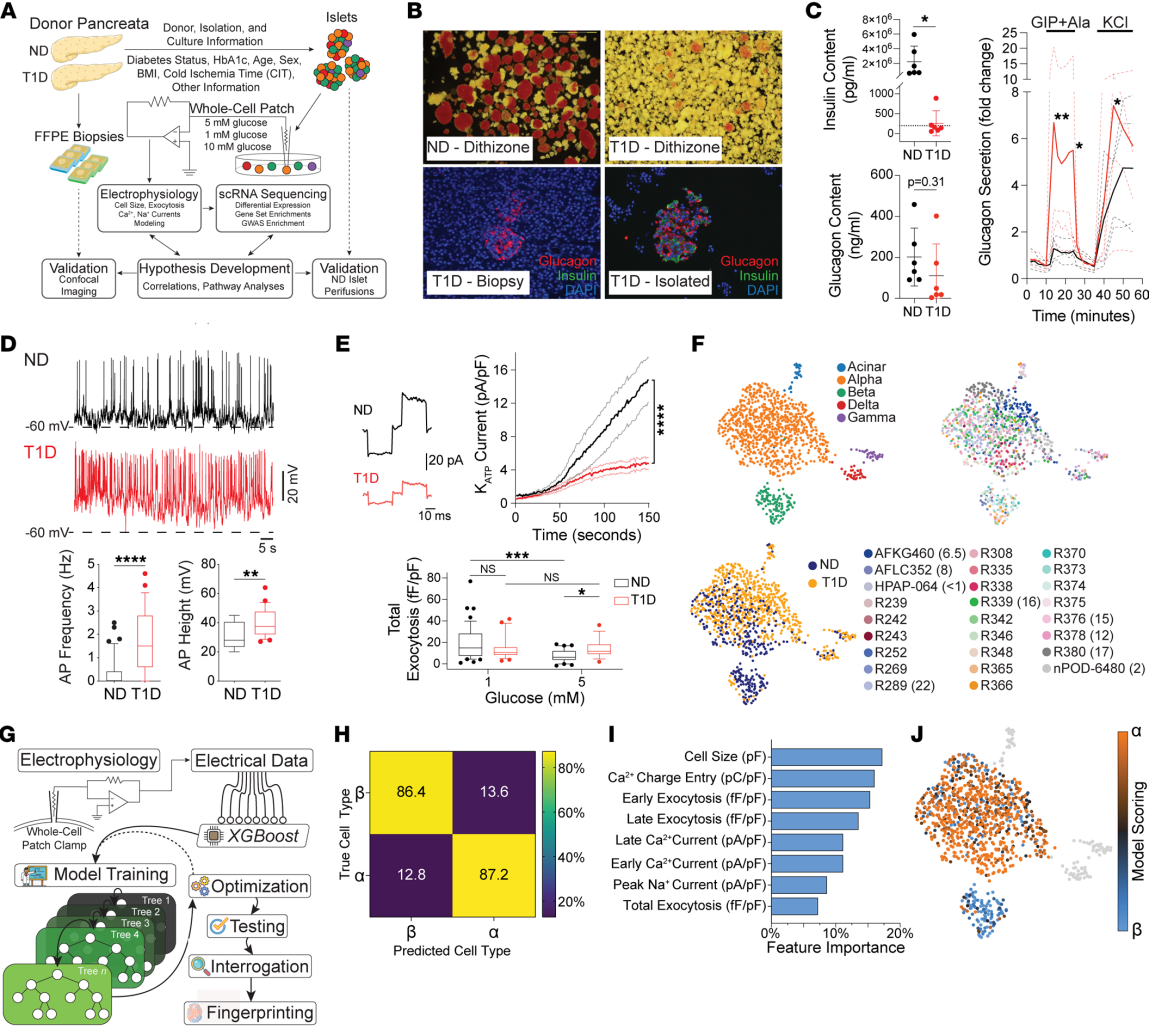
Patch-seq and electrophysiological profiling of donor islet cells. (**A**) Schematic of the patch-seq workflow: human islet isolation, single-cell dissociation, electrophysiology and transcriptomics, integrative modeling and validation. (**B**) ND and T1D islets stained with dithizone (red; 63x magnification) to identify regions with insulin, or immunofluorescence for glucagon (red), insulin (green), and nuclei (blue) from pancreatic biopsies or postisolation (200x magnification). (**C**) Glucagon and insulin content and glucagon secretion profiles of perifused islets from ND (*n =* 4) and T1D (*n =* 4) donors, showing elevated glucagon secretion upon stimulation with glucose-dependent insulinotropic polypeptide (GIP) and alanine (Ala). (**D**) Representative actional potential (AP) traces with frequency and amplitude quantification in 4 ND (*n =* 49, 17 respectively) and 4 T1D (*n =* 26, 22 respectively) α cells, indicating increased excitability. (**E**) K_ATP_ current traces and quantification in ND (*n =* 42, 4 donors) and T1D (*n =* 29, 2 donors) α cells, implying that the threshold for depolarization and activating secretion is lower in T1D α cells. Normalized total exocytosis at 1 mM (ND = 44, 8 donors and T1D = 24, 2 donors) and 5 mM glucose (ND = 31, 8 donors and T1D = 18, 2 donors). (**F**) Uniform Manifold Approximation and Projection (UMAP) of patch-seq cells from ND (*n =* 375, 17 donors) and T1D (*n =* 692, 9 donors) annotated by cell type, diabetes status, and donor ID with duration of T1D (years). (**G**) Schematic of electrophysiological fingerprinting using the machine learning classifier XGBoost, a gradient-boosted decision tree classifier trained on electrical data from ND α and β cells. It demonstrated admirable testing accuracy (**H**), without overreliance on any single electrical feature (**I**). The model analyzed the electrophysiology of our patch-seq data, providing a representative “score” for canonical α or β cell behavior, and is annotated onto the UMAP (**J**). Statistical tests: Mann-Whitney (**C**–**E**) or 2-way ANOVA (**C** and **E**). *P < 0.05; **P < 0.01; ***P < 0.001; ****P < 0.0001. Box and whiskers represent 10th–90th percentiles. Error bars are SD (**C**).

Consistent with previous studies (8, 11, 26), we isolated islets from organ donors with T1D (Figure 1A) and observed dithizone-positive tissue, albeit at lower levels, suggesting zinc-containing islets (Figure 1B). Most of these donors had long-standing T1D and are described in Supplemental Table 1 (supplemental material available online with this article; https://doi.org/10.1172/JCI195267DS1). α cell–enriched islets were present in T1D pancreatic tail biopsies and isolated islets, with sporadic β cells (Figure 1B). Insulin content was scarce, while glucagon content was not significantly reduced compared with age and sex-matched controls (Figure 1C). Insulin secretion was difficult to detect reliably (not shown), while glucagon secretion was hyperresponsive to stimuli (Figure 1C), consistent with increased glucagon responses to mixed meals in patients with T1D (20) and reports of increased responsiveness to cAMP-raising agents from isolated islets of autoantibody-positive donors (22). Isolated T1D α cells showed increased excitability, with higher action potential frequency and amplitude than nondiabetic (ND) controls at 5 mM glucose (Figure 1D), and lower ATP-sensitive K^+^ (K_ATP_) channel current density (Figure 1E). Unlike ND α cells, which showed a significant reduction in total exocytosis from 1 to 5 mM glucose, T1D α cell exocytosis was not suppressed by elevating glucose (Figure 1E).

We collected patch-seq data (16) from islet cells of donors with and without T1D and identified cell types via integration with a larger set of islet cell patch-seq data (16, 25) (Supplemental Table 1). Leiden clusters expressing *GCG*, *INS* and *IAPP*, *SST*, *PPY*, or *PRSS1* and *PRSS2,* grouped together in UMAP space, and were identified as α, β, δ, γ, and acinar cells, respectively (Supplemental Figure 1). Overlays of donor age, BMI, HbA1c (%), sex, diabetes status, and diabetes duration suggested good integration within cell types (Supplemental Figure 2). As organ donors with T1D are typically younger, subsequent analyses used controls matched for age, sex, BMI, and cold ischemic time (CIT) (Supplemental Figure 3). The resulting 1,067 cells from 17 ND and 9 T1D donors retained clustering by cell type (Figure 1F and Supplemental Figure 4), irrespective of diabetes status and showed good integration (Supplemental Figure 5).

To quantify canonical behavior, we performed electrophysiological fingerprinting (25, 27). We trained a machine learning model (Figure 1G) on a larger data set (α cells: 258, β cells: 290) of the matched ND electrophysiology data expanded with patched cells whose identities were confirmed by immunostaining for insulin or glucagon from www.humanislets.com (28). The model performs with approximately 85% accuracy in identifying α and β cells on electrophysiological data alone without trivial majority class prediction (Figure 1H), with balanced reliance on parameters (Figure 1I), and unaffected by donor characteristics (Supplemental Figure 6). This allowed the assignment of probabilities to α cell (α score) or β cell (β score) identities, indicative of their overall functional phenotypes (Figure 1J).

### Transcriptomic alterations and loss of normal function in T1D β and α cells.

**Figure 2:**
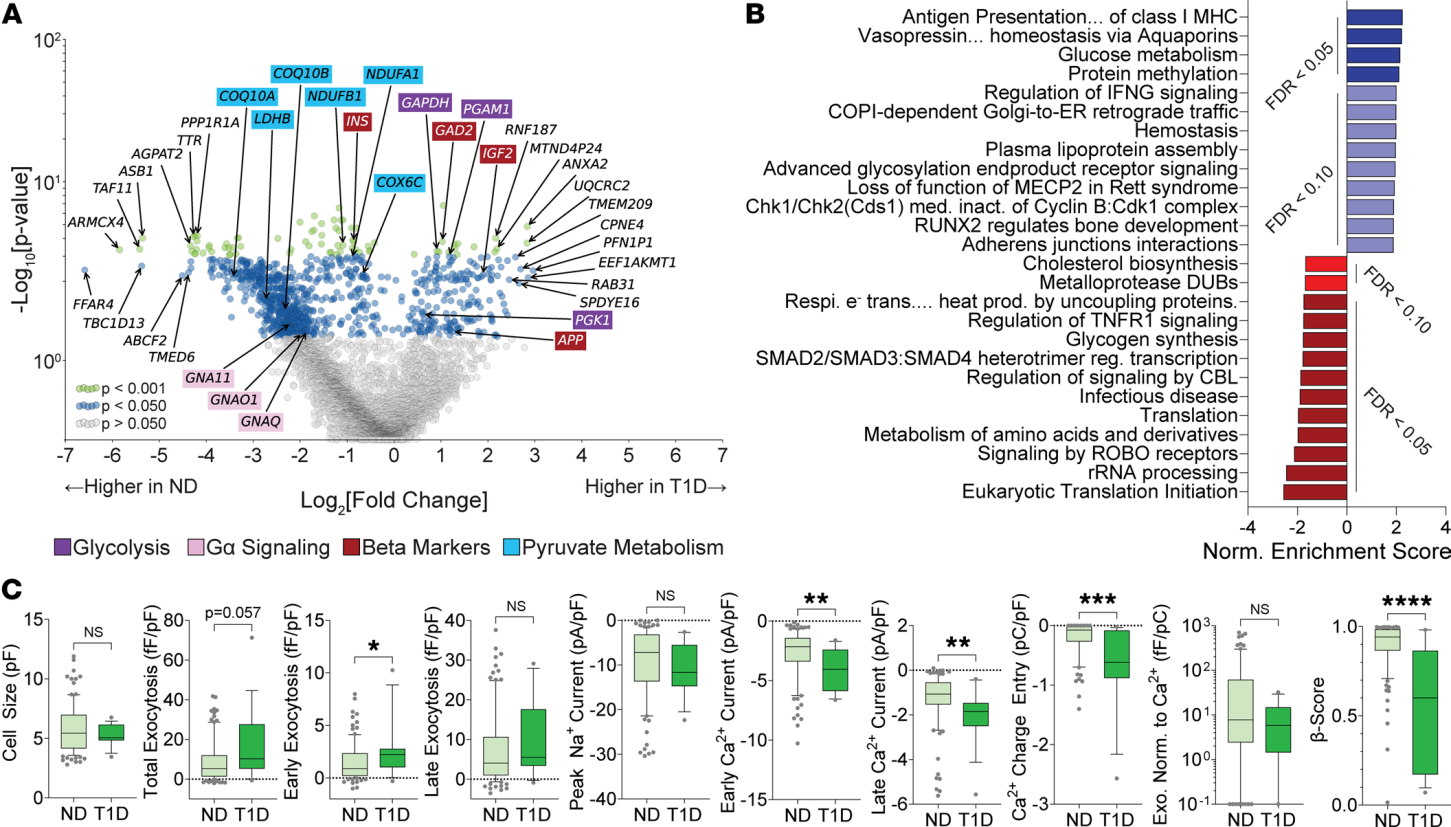
Patch-seq of T1D β cells at 5 mM glucose. (**A**) Volcano plot of differentially expressed transcripts in T1D (*n =* 16, 4 donors) versus ND β cells (*n =* 104, 14 donors). Notable transcript annotations include the top 10 up and downregulated genes (uncolored), glycolysis (purple), pyruvate metabolism (turquoise), Gα signaling (pink), and β cells (red). (**B**) Gene set enrichment analysis (GSEA) of transcripts from **A**, referenced against Reactome pathways. Upregulated pathways enriched in T1D are in blues, and downregulated pathways in reds. (**C**) Electrophysiological properties and model scores for β cells from ND (14 donors) and T1D (4 donors) recorded at 5 mM glucose: cell size (ND 104, T1D 16), total exocytosis (ND 102, T1D 16), early exocytosis (ND 102, T1D 15), late exocytosis (ND 102, T1D 15), peak Na^+^ current (ND 104, T1D 16), early Ca^2+^ current (ND 101, T1D 16), late Ca^2+^ current (ND 101, T1D 16), Ca^2+^ charge entry (ND 83, T1D 16), exocytosis/Ca^2+^ ratio (ND 80, T1D 16), and β-score (ND 104, T1D 16). Statistical tests: Mann-Whitney. *P < 0.05; **P < 0.01; ***P < 0.001; ****P < 0.0001. Outliers (|z|>3) excluded. Box and whiskers represent 10th–90th percentiles.

While T1D β cells were few (*n* = 16, 4 donors), they possessed differing transcriptomic and functional profiles compared to ND (*n* = 104, 14 donors) at 5 mM glucose. Differential expression analysis (DEA) and subsequent Gene Set Enrichment Analysis (GSEA) (Supplemental Table 2) revealed markers (Figure 2A) and pathways (Figure 2B) that include expected findings such as upregulation of antigen presentation related to MHC-I (29, 30) and IFN-γ (31), and downregulation of protein translation (30). Notably, T1D β cells show enrichment of markers (Figure 2A) and pathways (Figure 2B) for glycolysis (30) (Figure 2A, purple), and downregulation in pyruvate-related metabolism (Figure 2A, blue) and mitochondrial respiration (Figure 2B), suggesting a shift toward nonoxidative glucose metabolism. Other upregulated transcripts included *GAD2*, i.e. T1D autoantibody target GAD65 (32), *IGF2,* whose enrichment induces β cell dysfunction (33), and amyloid markers *IAPP* and *APP,* both implicated in T1D (34) and influencing islet behavior (35). Insulin secretion from T1D β cells is reported as either normal (7, 8) or impaired (11, 26), with function potentially restored after prolonged culture (12). However, we observed T1D β cells showing increased exocytosis and inward (i.e., more negative) voltage-activated Ca^2+^ currents with significant decreases in β score, indicating substantially altered behavior (Figure 2C).

**Figure 3:**
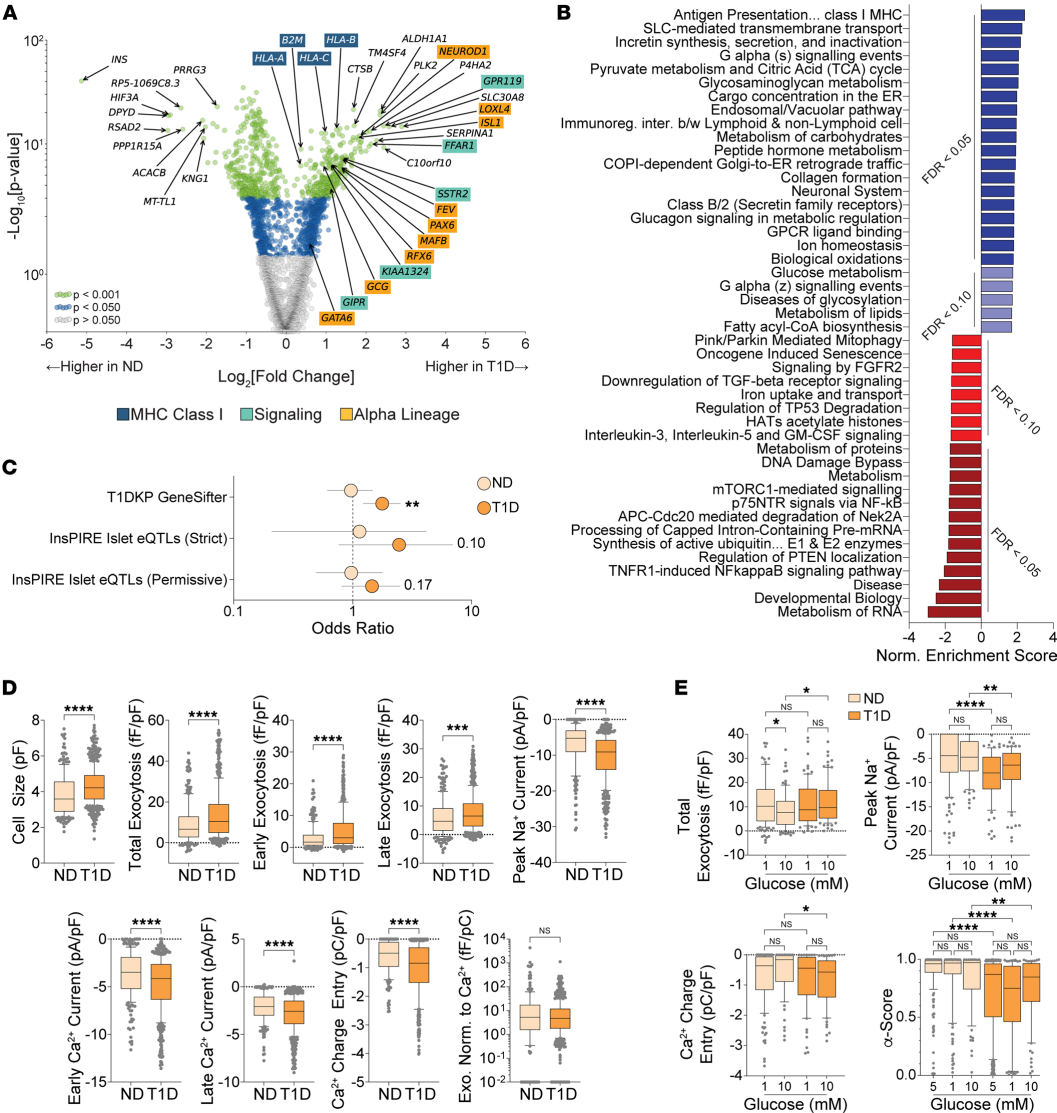
Patch-seq of T1D α cells. (**A**) Volcano plot of differentially expressed transcripts in T1D (*n =* 596, 9 donors) versus ND (*n* = 248, 17 donors). Notable transcript annotations include the top 10 up and downregulated genes (uncolored), MHC class I antigen presentation (blue), signaling (turquoise), and α cell lineage (orange). (**B**) Gene set enrichment analysis (GSEA) of transcripts from **A**, referenced against Reactome pathways. Upregulated pathways enriched in T1D are in blues, and downregulated pathways in reds. (**C**) Odds ratios showing enrichment of T1D risk genes among differentially expressed transcripts in T1D α cells. Risk genes were derived from the T1D Knowledge Portal and islet eQTLs. Error bars represent 95% confidence intervals. (**D**) Electrophysiological properties for α cells from ND (17 donors) and T1D (9 donors) recorded at 5 mM glucose: cell size (ND 245, T1D 585), total exocytosis (ND 243, T1D 588), early exocytosis (ND 241, T1D 583), late exocytosis (ND 246, T1D 584), peak Na^+^ current (ND 238, T1D 577), early Ca^2+^ current (ND 236, T1D 580), late Ca^2+^ current (ND 236, T1D 577), Ca^2+^ charge entry (ND 205, T1D 531), exocytosis/Ca^2+^ ratio (ND 195, T1D 529). (**E**) Electrophysiological properties for α cells at differing glucose concentrations. At 1 mM glucose, ND (13 donors) and T1D (4 donors): cell size (ND 156, T1D 81), total exocytosis (ND 154, T1D 81), peak Na^+^ current (ND 154, T1D 78), Ca^2+^ charge (ND 149, T1D 75). At 10mM glucose, ND (10 donors) and T1D (4 donors): cell size (ND 85, T1D 80), total exocytosis (ND 84, T1D 80), peak Na^+^ current (ND 84, T1D 81), Ca^2+^ charge entry (ND 77, T1D 70). Model scoring of α cells from ND (17, 13, 10 at 5, 1, and 10 mm, respectively) and T1D (9, 4, 4 at 5, 1, and 10 mm, respectively) donors, at glucose concentrations of 5 mM (ND 248, T1D 596) 1 mM (ND 158, T1D 82) and 10 mM (ND 85,T1D 81). Statistical tests: Fisher’s Exact Test (**C**). Mann-Whitney (**D**). Kruskal-Wallis with Dunn’s correction (**E**). *P < 0.05; **P < 0.01; ***P < 0.001; ****P < 0.0001. Outliers (|z|>3) excluded. Box and whiskers represent 10th–90th percentiles.

Similarly, T1D α cells (*n* = 596, 9 donors) are enriched (Supplemental Table 2) in antigen presentation markers (Figure 3A, blue) and pathways (Figure 3B) related to MHC-I compared with control (*n* = 248, 17 donors). We also observed an increase in pathways related to incretin secretion (36). Our work in type 2 diabetes (T2D) revealed α cell dysfunction linked to maturity state (25), and we observe an enrichment of similar markers in T1D α cells (Figure 3A, orange), corroborating previous findings (24). Paracrine receptors that modulate glucagon secretion are also upregulated (Figure 3A, turquoise)**,** including the GIP receptor (*GIPR*), with enrichments in Gα (s) and GPCR signaling (Figure 3B). Additionally, pathways for gluconeogenesis, carbohydrate metabolism, TCA cycle, and lipid metabolism are enriched in T1D α cells (Figure 3B). Downregulated pathways included cytokine-related pathways and mTORC1 (Figure 3B). We further detected the enrichment of transcripts differentially expressed in T1D α cells among genes with T1D genetic association, as well as genes directly regulated by T1D-associated variants from islet expression QTL (eQTL) data (Figure 3C).

T1D α cells were significantly larger and showed greater exocytosis with increased Ca^2+^ and Na^+^ currents at 5 mM glucose (Figure 3D), indicating broad alterations in phenotypes of enhanced excitability, as observed in Figure 1, D and E, and complemented by decreased α scores, suggesting a loss of canonical behavior (Figure 3E). As glucose impacts α cell secretion (25, 37), we confirmed reduced exocytosis and Ca^2+^ currents in ND α cells at high glucose (10 mM); however, T1D α cells fail to show these glucose-mediated changes, while also demonstrating elevated Na^+^ currents (Figure 3E and Supplemental Figure 7). Further, T1D α cells showed significantly lower α scores, irrespective of glucose concentration, implying a consistent loss in behavior (Figure 3E and Supplemental Figure 7).

### Transcripts and pathways linked to T1D α cell function.

**Figure 4:**
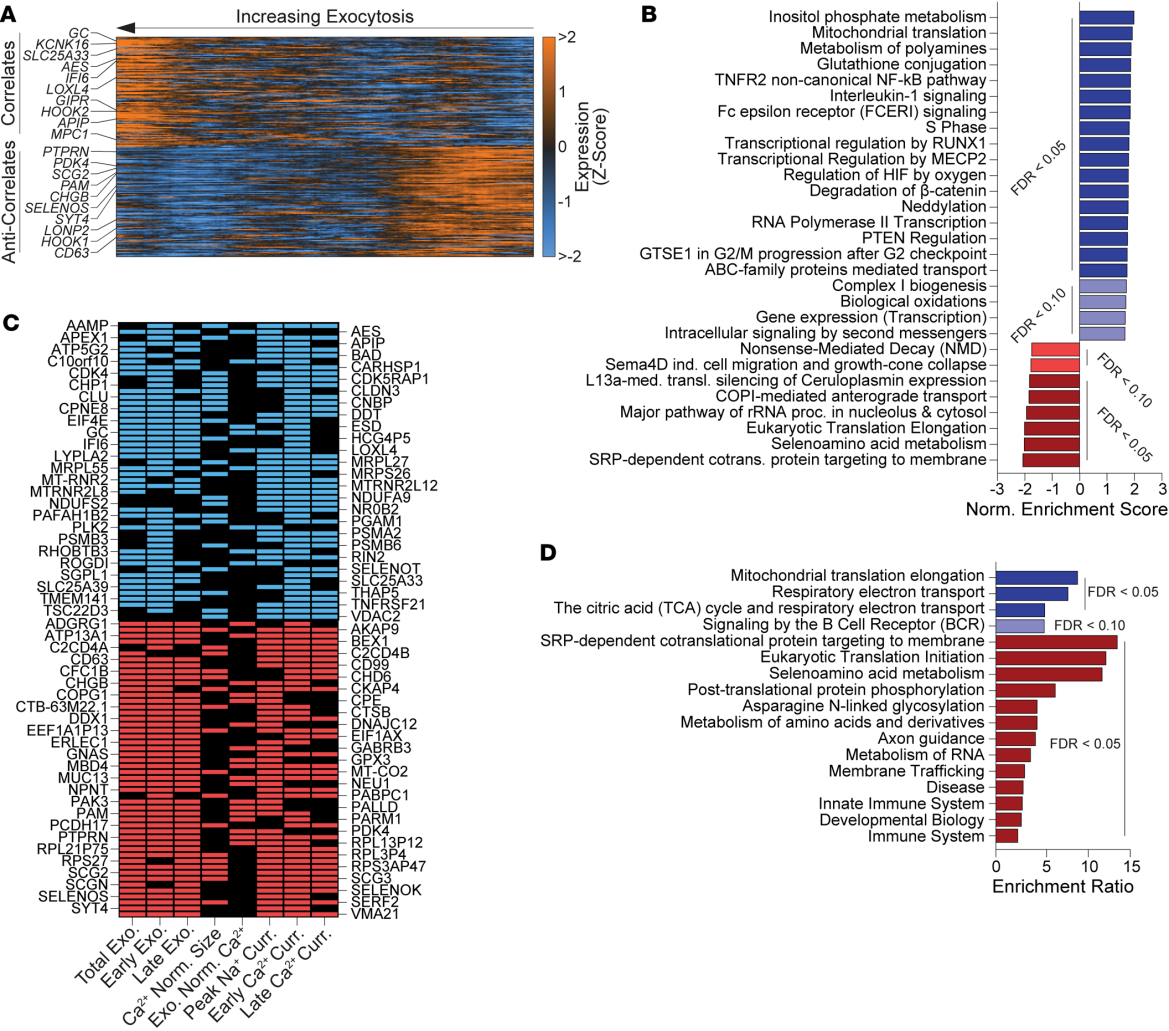
Linking transcript expression with electrophysiology in T1D patch-seq α cells at 5mM glucose. (**A**) Expression heatmap (as Z-score with smoothing of *n =* 50) of top and bottom T1D α cell transcript correlates of normalized total exocytosis. (**B**) Correlating (blues) and anticorrelating (reds) reactome pathways from the gene set enrichment analysis of T1D α cells using transcript–exocytosis correlation coefficients. (**C**) Top significant transcripts correlating (blue) or anticorrelating (red) with secretions across ≥ 3 properties. (**D**) Correlating (blues) and anticorrelating (red) Reactome pathways from the over representation analysis of significant transcripts trending across ≥ 3 properties.

We identified pathways in T1D α cells linked to their functional deficits by correlating transcript expression with exocytosis (Supplemental Table 3). Among the top significant correlates (Figure 4A) were transcripts previously associated with α cell behavior like *GC* (vitamin D binding protein) (38) and *LOXL4* (lysyl oxidase like 4) (16), with the latter also contributing to immune evasion (39). The IFN effector *IFI6* (IFN-α inducible protein 6) (40), which increases in T1D (41), also positively correlated with exocytosis. Other correlates previously linked to increased glucagon secretion included the 2-pore–domain K^+^ channel *KCNK16* (TALK-1) (42), *GIPR* (43), and *MPC1* (Mitochondrial Pyruvate Carrier 1) (44). Anticorrelates included *PDK4* (Pyruvate Dehydrogenase Kinase 4) (44), which regulates glucagon secretion (45), and *HOOK1* (Hook Microtubule Tethering Protein 1) (46), which may mediate endolysosomal glucagon degradation, lowering secretory capacity (47). Other anticorrelates include *SYT4* (Synaptotagmin 4), which reduces secretion by competing with prosecretory Synaptotagmin 7 (48), and stress-relief markers, like *SELENOS* (Selenoprotein S) (49) and *LONP2* (Lon Peptidase 2) (50).

We next identified pathways correlating with T1D α cell exocytosis (Figure 4B and Supplemental Table 3), which reflect immune influences on α cell exocytosis. Positive correlates included interleukin-1 signaling (51), and TNFR2 noncanonical NF-kB pathways, which parallel the chronic nature of T1D (52). Most transcripts contributing to the enrichment of these pathways were proteasomal components, which upregulate during inflammation (53). Finally, we identified transcripts that correlated across multiple electrophysiological parameters (Figure 4C and Supplemental Table 4), with the subsequent over-representation analysis (ORA) (Figure 4D and Supplemental Table 4), suggesting mitochondrial respiration as a contributor to altered behavior, previously seen in T2D α cell dysfunction (25).

### Linking electrophysiological phenotypes to molecular pathways and genetic risk in T1D α cells.

**Figure 5:**
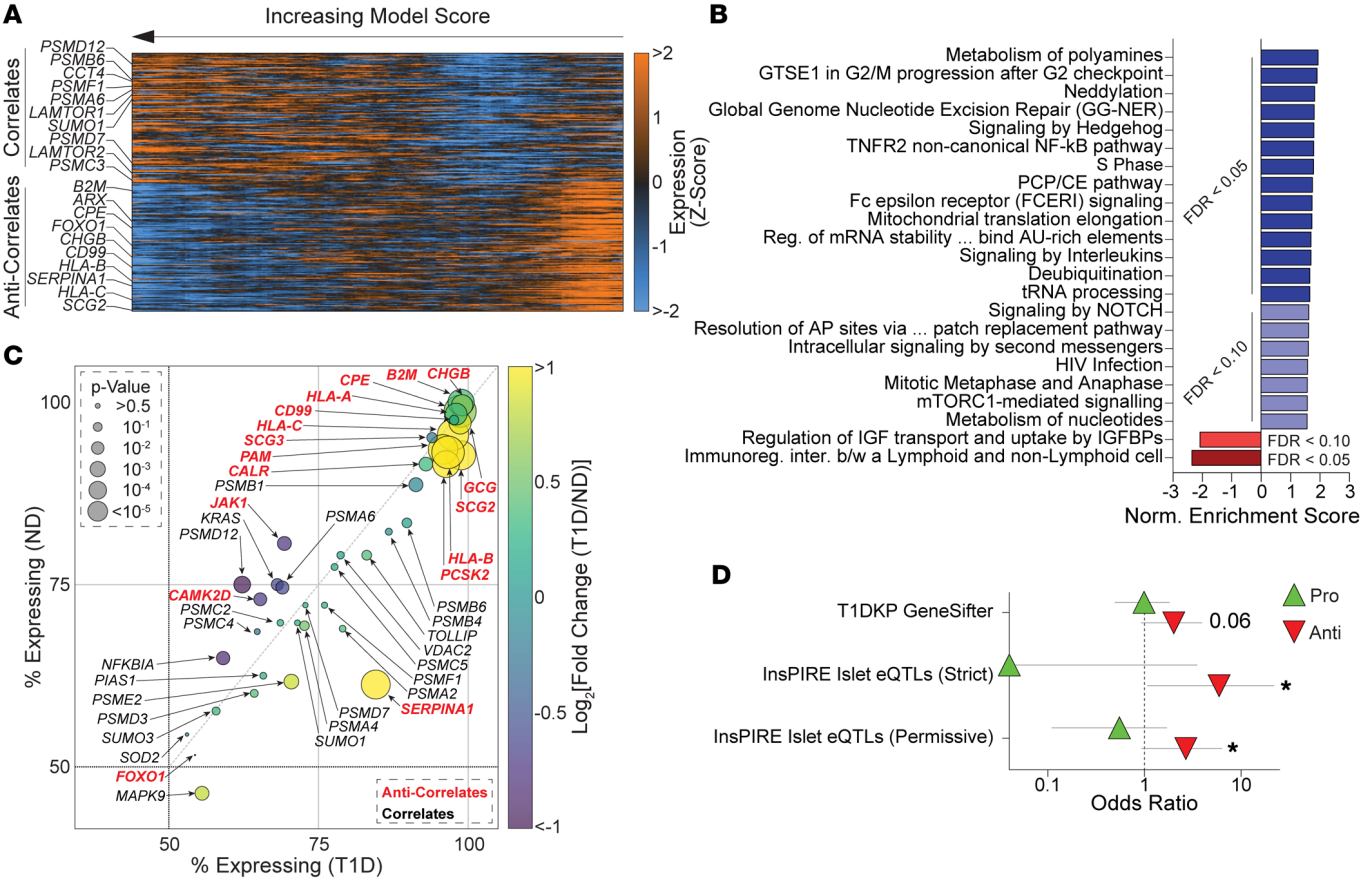
Linking transcript expression with model scoring in T1D patch-seq α cells at 5 mM glucose. (**A**) Expression heatmap (as Z-score with smoothing of (*n =* 50) of top and bottom T1D α cell transcript correlates of model (α) score. (**B**) Correlating (blues) and anticorrelating (reds) reactome pathways from the gene set enrichment analysis of T1D α cells using transcript–score correlation coefficients. (**C**) Bubble plot of notable transcript–score correlates (black) and anticorrelate (red) in T1D α cells, with their differential and percent expression in ND and T1D. (**D**) Odds ratios for enrichment of T1D risk genes among transcripts that correlate (Pro, green triangles) or anticorrelate (Anti, red triangles) with α score in T1D α cells. Risk genes and T1D-associated variants were compiled from the T1D Knowledge Portal and islet eQTL datasets. Error bars indicate 95% confidence intervals. Statistical tests: Fisher’s Exact Test. *P < 0.05.

While exocytosis represents vesicle-mediated secretion, α scores provide an integrated metric for overall behavior (Figure 1, G–J). Top correlates with α score in T1D (Figure 5A and Supplemental Table 4) included proteasomal components (*PSMA6*, *PSMB6, PSMF1, PSMD12, PSMD7,* and *PSMC3)*, suggesting that canonical cell behavior in T1D may rely on higher proteasomal activity, likely associated with alleviating ER stress and unfolded protein responses (24). Positive correlates also included mTORC1 assembly components *LAMTOR1* and *LAMTOR2* (Late Endosomal/Lysosomal Adaptor, MAPK and mTOR Activator 1, 2) (54), and *CCT4* (Chaperonin Containing TCP1 Subunit 4), which regulates lysosome acidification (55). Further, *SUMO1* (Small Ubiquitin Like Modifier 1), which influences cAMP-mediated exocytosis (56) and suppresses NF-κB immune signaling by protecting its inhibitor IκBα (*NFKBIA)* (57), also positively correlated with α-score. Expectedly, negative correlates with α score were MHC-I antigen presentation (*B2M, HLA-B, HLA-C)* and the α cell transcription factor (TF) *ARX* (Aristaless Related Homeobox) with its enhancer *FOXO1* (58) (Forkhead Box O1), previously associated with α cell dysfunction in T2D (25).

GSEA of the α-score correlations (Figure 5B and Supplemental Table 4) revealed pathways containing proteasomal transcripts, and mTORC1-mediated signaling, whose components were previously detected as top trending correlations (Figure 5A). Positive correlating pathways also included suppressors of immune signaling effectors such as *PIAS1* (Protein Inhibitor of Activated STAT1) (59), *NFKBIA* (57), and *TOLLIP* (Toll Interacting Protein) (60) (Supplemental Table 4). Pathways anticorrelating with α score involved immune signaling consisting of antigen presentation components (*HLAs, B2M*) and β cell dedifferentiation marker *CD81* (TSPAN28) (61). To investigate the relevance of the T1D α-score correlate hits, we plotted their differential expression (Figure 5C), revealing that most negative correlates showed significantly higher expression in T1D (Figure 5C, *red*), contrasting with the positive correlates (Figure 5C, *black*). Next, we cross referenced the correlates against genes with evidence for T1D genetic association and discovered that negative correlates of α-score overlap with genes directly regulated by T1D risk variants in islet eQTL data (Figure 5D); thus, linking genetic predispositions, variations in transcriptomic expression, and cellular dysfunction. Finally, we categorized intriguing correlative hits by function and integrated their expression patterns to determine candidate drivers of α cell dysfunction in T1D for experimental validation (Supplemental Figure 8 and Supplemental Table 5).

### Altered MHC-I localization and reduced nuclear presence of α cell lineage factors in T1D.

**Figure 6:**
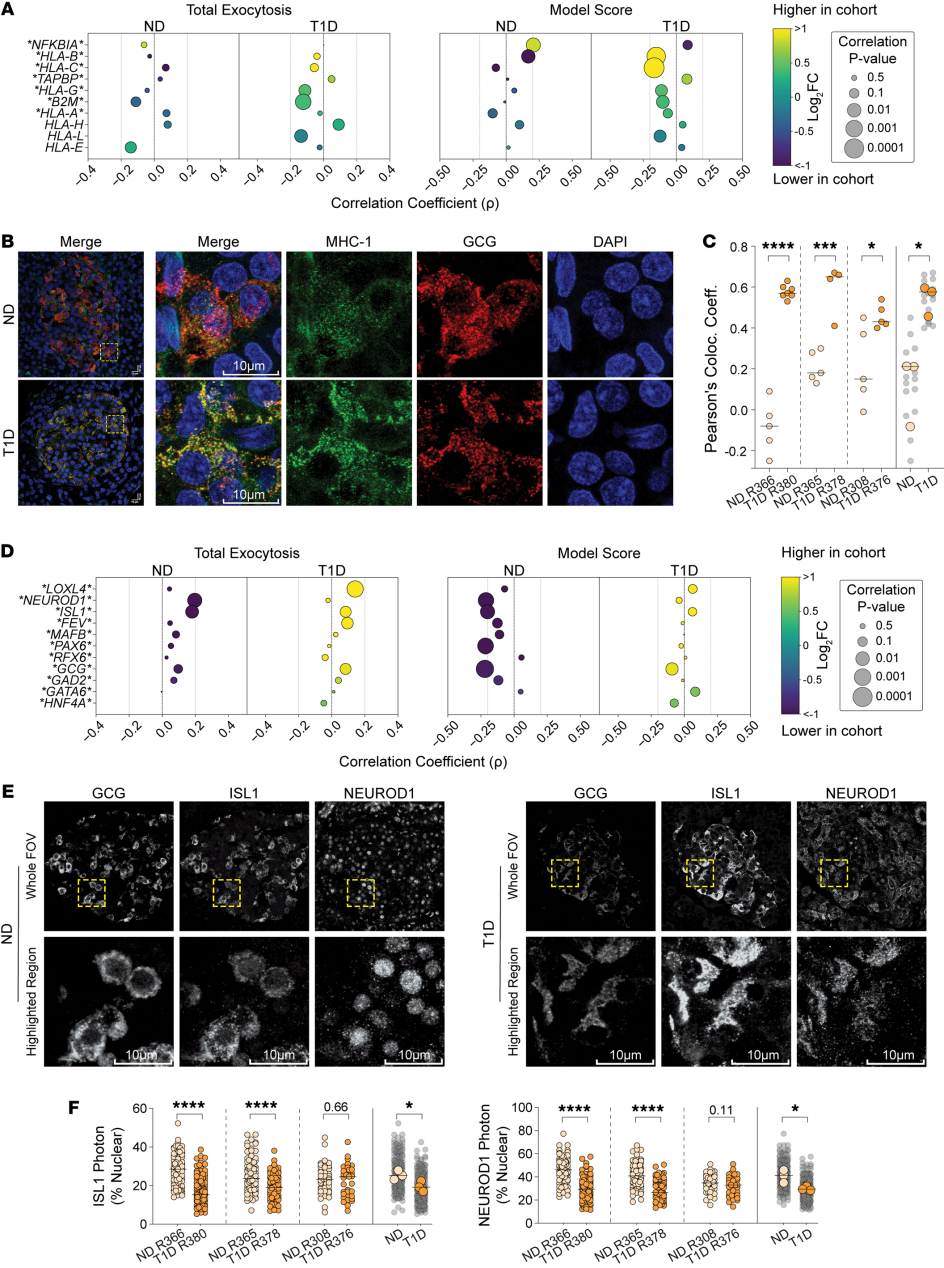
Altered localizations of MHC-I, and transcription factors ISL1 and NEUROD1 in T1D α cells. (**A**) Combined differential expression and correlative analysis of notable markers of MHC-I presentation in α cells at 5 mM glucose (excerpt from Supplemental Figure 8). Asterisks indicate significant differential expression. (**B**) Representative confocal immunofluorescence images of MHC-I (green) in pancreas biopsies of patch-seq donors, showing colocalization (yellow) with glucagon (GCG, red) in T1D. (**C**) Quantification of MHC-I and glucagon colocalization. Each point represents a single islet field of view in ND (*n* = 15, 3 donors) and T1D (*n* = 16, 3 donors) biopsies. (**D**) Combined DEA and correlation analysis of notable markers of α cell lineage in α cells at 5 mM glucose (excerpt from Supplemental Figure 8). Asterisks indicate significant differential expression. (**E**) Representative confocal immunofluorescence images of GCG, ISL1, and NEUROD1 in pancreas biopsies of patch-seq donors, in greyscale for improved contrast. (**F**) Quantification of ISL1 and NEUROD1 nuclear presence. Each point represents single cells from multiple islets in ND (*n =* 231, 3 donors) and T1D (*n =* 231, 3 donors) biopsies. Statistical tests: Unpaired 2-tailed *t* test. *P < 0.05, ***P < 0.001, and ****P < 0.0001. Horizontal lines represent median.

The combined DEA and correlative analysis of MHC-I–related transcripts is presented in Figure 6A. NF-κB drives MHC-I presentation (62), and its inhibitor *NFKBIA* is downregulated in T1D, yet positively associates with α score. Contrastingly, MHC-I transcripts are upregulated in T1D and consistently trend with worsening α scores. Glucagon maturation and exocytosis is dependent on secretory vesicle trafficking in α cells (47), processes shared by MHC-I processing and surface presentation (63). Also, α cells display enriched MHC-I expression (23), upregulated further in T1D (24) and aging-related cellular stress (64). 3D confocal microscopy of pancreas sections showed higher colocalization of glucagon, with MHC-I in T1D donor biopsies compared with matched controls (Figure 6, B and C), suggesting that MHC-I upregulation could impact glucagon’s secretory pathway. Immune-related signal transducers like NF-κB and STATs can monopolize import machinery, altering homeostatic nuclear permeability (65) and possibly impairing access for TFs dictating α cell identity. Indeed, we detect reduced expression of α importins *KPNA4* and *KPNA6*, as well as β importins *KPNB1* and *TNPO1* (Supplemental Table 5). Moreover, *KPNA4*, *KPNA6*, and *TNPO1* are significantly associated with improved model scores in ND, an association that is absent in T1D (Supplemental Table 5). This prompted us to examine whether TFs critical for α cell identity exhibit altered nuclear localization. The upregulation of α cell lineage markers like TFs *ISL1* and *NEUROD1* were reported in T1D (24), and, in T2D, associated with α cell dysfunction (25). These transcripts trend with exocytosis but only associate with lower α-scores in ND (Figure 6D). Indeed, such TFs upregulate to drive (re)maturation in α cells, however, this feedback is likely lost in the T1D α cells, which show dysfunction despite their upregulation. Thus, we evaluated the subcellular localization of ISL1 and NEUROD1 in T1D α cells. Despite heterogeneity within and between donors, nuclear localization was higher in ND compared with T1D (Figure 6, E and F). Moreover, regardless of diabetes status, nuclear ISL1 levels trended with nuclear NEUROD1 but were overall reduced in T1D (Supplemental Figure 9), suggesting that, despite upregulation, a general impairment of nuclear access, potentially stemming from a reduction in importins, prevents α cell (re)maturation in T1D.

### Reduced mTORC1 and lysosomal disorder in T1D α cells.

**Figure 7:**
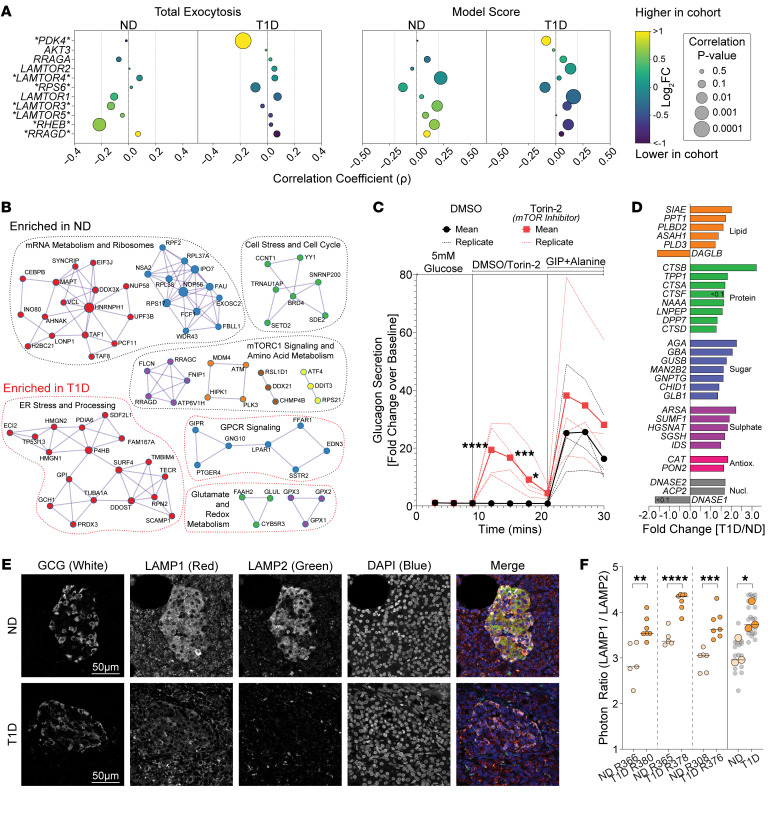
Suppressed mTOR enhances glucagon secretion and affects lysosomal balance in T1D α cells. (**A**) Combined differential expression and correlative analysis of notable markers of mTORC1 signaling in α cells at 5 mM glucose (excerpt from Supplemental Figure 8). Asterisks indicate significant differential expression. (**B**) Protein-protein interaction networks of differently expressed genes enriched in ND (black outlines) or T1D (red outlines) α cells. (**C**) Effects of mTOR inhibition by 1 μM Torin-2 on glucagon dynamic secretion perfusion in ND islets (4 donors). (**D**) Expression of significant differentially expressed lysosomal hydrolytic enzymes in T1D α cells compared with ND. (**E**) Representative confocal immunofluorescence images of glucagon (GCG), LAMP1, and LAMP2 in pancreas biopsies of patch-seq donors. Single-channel images are shown in greyscale; labels indicate color in the merged image. (**F**) Quantification of LAMP1/LAMP2 in α cells. Each point represents a single islet field of view in ND (*n* = 16, 3 donors) and T1D (*n* = 21, 3 donors) biopsies. Statistical tests: Unpaired 2-tailed *t* test (**F**). Two-way ANOVA (**C**). *P < 0.05, **P < 0.01, ***P < 0.001, and ****P < 0.0001. Horizontal lines represent median.

The mTORC1 pathway repeatedly emerged across analyses, downregulated in T1D α cells (Figure 3E) yet positively associated with α score (Figure 5B). Components of mTORC1 correlate with lower exocytosis in ND — a relationship lost in T1D — but still maintain positive trends with α score regardless of diabetes status, suggesting their importance for canonical behavior (Figure 7A). However, these components are downregulated in T1D α cells (Figure 7A). Protein-protein interaction network analyses corroborated that amino acid metabolism and regulatory components of mTORC1 activity were significantly abrogated as well (Figure 7B). While mTORC1 influences myriad pathways in sensing nutrients (66) and regulating lysosomes (55), it is also influenced by insulin receptor signaling (67). Considering the near absence of β cells and associated insulin in the T1D islet microenvironment, we next explored mTORC1’s contribution to α cell dysfunction. To mimic mTORC1 downregulation in T1D α cells and its impact on glucagon secretion, we treated ND islets with an inhibitor, Torin-2, and performed dynamic secretion assays at 5 mM glucose (Figure 7C). Glucagon secretion, and, to a lesser extent, insulin (not shown), were increased upon Torin-2 administration, suggesting that reduced mTORC1 signaling contributed to hypersecretion. Next, we investigated lysosomes, which are regulated by mTORC1, and implicated in diabetes pathophysiology (68). Lysosomal-associated hydrolytic enzymes (69) were upregulated in T1D α cells (Figure 7D). Moreover, α cells exposed to hyperglycemia showed that glucagon degradation at LAMP2^+^ lysosomes is reduced, with increased redirection to secretory LAMP1^+^ lysosomes (47). Imaging LAMP1 and LAMP2 (Figure 7E) revealed lower signals in T1D α cells, but a higher LAMP1/LAMP2 ratio, suggestive of lysosomal disorder (Figure 7F).

## Discussion

Research on isolated islets from donors with T1D is impacted by the rarity of acquiring human tissue for study. Single-donor studies suggest a loss of glucose-dependent insulin secretion when isolated at (26) or months after (11) diagnosis, but perhaps some glucose-responsive secretion in long-standing T1D (8). Moreover, glucose suppression of glucagon secretion appears lost, with preserved arginine responses (8, 11). A multidonor study on islets from 8 donors with T1D suggested normal glucose-dependent insulin secretion of remaining β cells but disrupted glucagon secretion (7). And islets from 6 donors with T1D (3 included in the present study), showed glucagon secretion that was unresponsive to glucose but increased with amino acids (70). Furthermore, somatostatin secretion from T1D islets was increased, and the antagonism of this increase also led to increased glucagon secretion. Alterations in glucagon secretion appear to occur early in T1D, possibly even before clinical diagnosis, given the differences observed in islets from 9 donors without T1D but positive for GADA autoantibody (22).

We find significant differences in the phenotypes of α and β cells from donors with T1D compared with ND controls matched for age, sex, and BMI. Electrophysiological distinctions were demonstrated by altered activity of key ion channels, exocytotic processes, and aggregate biophysical properties (i.e., the α/β scores). This loss of electrophysiologic phenotype likely contributes to observed dysregulated function, such as the increased α cell excitability and responsiveness to stimuli. By connecting transcriptional changes with biophysical phenotypes, we not only captured known transcriptional changes (e.g., MHC-I upregulation) but also revealed genes and pathways likely contributing to α cell dysfunction in T1D. Furthermore, these transcripts, linked to exocytotic dysfunction and loss of functional phenotypes in T1D α cells, are enriched for genes involved in T1D risk. We validated some of these candidates of α cell dysfunction, showing altered MHC-I and glucagon localization, a reduction in nuclear abundance of α cell lineage TFs ISL1 and NEUROD1, and potential roles of suppressed mTOR activity and disordered lysosomal homeostasis.

Studies of live pancreas slices from donors with T1D show that remaining β cells are functionally impaired in T1D (9, 13, 14), which is consistent with the loss of electrophysiological phenotype in our findings and the transcriptomic upregulation of glycolysis and downregulation of mitochondrial respiration, suggesting a “stem-like” metabolic phenotype (71). Indeed, the metabolic interplay between glucose metabolism and mitochondrial respiration observed in stem cell–derived β cells is a driving factor in their altered secretory behavior (72, 73). Overall, we provide patch-seq evidence that the remaining β cells in T1D have altered functional and transcriptomic phenotypes. However, due to their limited availability, we focused on a deeper analysis of α cell phenotypes.

Glucagon secretion is dysregulated in T1D, lacking stimulation by hypoglycemia (19) and an inappropriately elevated response to a mixed meal (20), while in the isolated islets, glucagon stimulation by low glucose appears impaired, but not to arginine (7, 8, 11, 74), although this finding is not universal (14). Interestingly, glucagon secretion from islets of autoantibody-positive donors are hyperresponsive to cAMP-raising agents (22). Consistent with this, we find that islets of T1D donors are hyperresponsive to GIP and alanine. GPCR signalling (including *GIPR* expression) appears upregulated and correlates with inappropriately elevated exocytosis in T1D α cells, complementing the elevated serum levels of GIP reported in T1D patients (75). The survival of α cells in T1D is somewhat enigmatic (76). One paradigm is that α cells better handle ER stress and unfolded protein responses (76) and show greater resistance to apoptosis (77), which may be evident by the upregulation of proteasomal components that correlate with α score to a greater extent in T1D than in ND, and the upregulation of markers like *BCAP31, BCL2L1,* and *APIP,* respectively (Supplemental Figure 8). Further complicating their autoimmune evasion in T1D, α cells in ND not only possess elevated MHC-I (23) but may also contribute to macrophage and T cell recruitment (78). Indeed, we see MHC-I (*HLA-A/B/C* and *B2M)* elevated in T1D α cells and correlating with worsening α scores while those expressing higher levels of immune signaling inhibitors (*PIAS1, TOLLIP,* and *NFKBIA)* associate with improved α scores. The microscopy showing compartmentalization of GCG and MHC-I in ND α cells, but colocalization in T1D, suggests the possibility of upregulated antigen presentation contributing to dysfunction by impacting the glucagon secretory pathway. Thus, in T1D, α cell susceptibility to dysfunction via MHC-I upregulation could be due to a combination of cell-intrinsic and -extrinsic influences. Cytokine exposure (79) may be variable across the T1D pancreatic environment (80, 81), while intrinsically higher immune inhibitory signals within some α cells could help preserve canonical α cell behavior.

Markers of α cell lineage (82), such as TFs *PAX6,* *NEUROD1,* and *ISL1*, have been shown as upregulated in T1D α cells (24), and our analyses corroborate these findings. We also see that, in ND α cells, higher expression of these markers correlate with a decrease in α score, which may be unsurprising as a subset of α cells likely possess altered functional behavior due to immaturity, or a transient loss of maturity, which would then drive the expression of these markers (83). However, the correlation is mostly lost in the T1D α cells, suggesting a potential breakdown in this feedback mechanism. Microscopy revealed reduced nuclear NEUROD1 and ISL1 in T1D α cells, suggesting that, despite upregulation, impaired nuclear access may limit their ability to restore α cell maturity. In fact, immune signaling (and viral infections) can monopolize nuclear transport machinery, leading to a disruption of homeostatic nuclear permeability (65), and, in conjunction, we also found reduced expression of nuclear import machinery members *KPNA4, KPNA6,* and *TNPO1* associated with α cell dysfunction.

Previous studies showed that prolonged hyperglycemia sustains mTORC1 activity in α cells, leading to elevated glucagon secretion (84). Inhibition of mTOR, either via α cell–specific RAPTOR knockout (84, 85) or rapamycin treatment (85), abrogates this secretion. Notably, RAPTOR knockouts were conducted in mice, and rapamycin was administered chronically, which can deplete ER and mitochondrial Ca²^+^ stores, impairing mitochondrial respiration and reducing secretion (86). Here, we report that during euglycemia (5 mM glucose) acute mTOR inhibition using Torin-2 — a potent and selective inhibitor — increases glucagon secretion from ND islets. mTORC1 components positively correlate with α score, supporting their role in α cell function, but show a strong negative correlation with exocytosis in ND, a relationship lost in T1D. When considered alongside the significantly reduced expression of assembly/regulatory components in T1D α cells, including *RHEB*, *LAMTOR*s, and *RRAGD*, these findings imply that reduced mTORC1 signaling contributes to dysfunction. This may further align with our earlier implication that MHC-I upregulation disrupts glucagon secretion, as impaired mTOR signaling induces MHC-I overexpression (87). Alterations in mTORC1 signaling in T1D α cells may also impact lysosomes. Studies in αTC1-6 cells — a model of α cells — show that, under chronic hyperglycemia, glucagon granules misroute from degradative LAMP2^+^ lysosomes to LAMP1^+^ secretory lysosomes, contributing to hypersecretion (47). This complements our observations of elevated secretory responses and increased LAMP1/LAMP2 ratios in T1D α cells. While a full mechanistic analysis is beyond the scope of this study, our findings suggest that T1D α cell dysfunction may involve coordinated alteration to mTORC1 signaling, lysosomes, and proteasomes. Notably, free amino acid pools link these pathways, and altered plasma amino acid profiles are a well-established feature of T1D (88).

While T1D genetic risk is strongly associated with T-cell activity, risk variants have also been linked to β cells and other pancreatic cell types, including acinar and ductal cells (89). Our findings now extend this to α cells, as genes upregulated in T1D α cells are enriched for T1D risk variants. This supports a model where genetic, transcriptomic, and biophysical factors interact with α cell dysfunction. Furthermore, T1D-associated genes were enriched among transcripts negatively correlated with α score and depleted among positive correlates in T1D α cells. These data suggest that α cells with more pronounced electrophysiological impairment (i.e., lower α scores) express higher levels of T1D risk genes, while cells with preserved function show reduced expression. Notably, MHC-I genes — key factors of T1D susceptibility — were among the most enriched. Together, these findings highlight a potential mechanistic link between α cell dysfunction and genetic predisposition to T1D.

**Figure 8:**
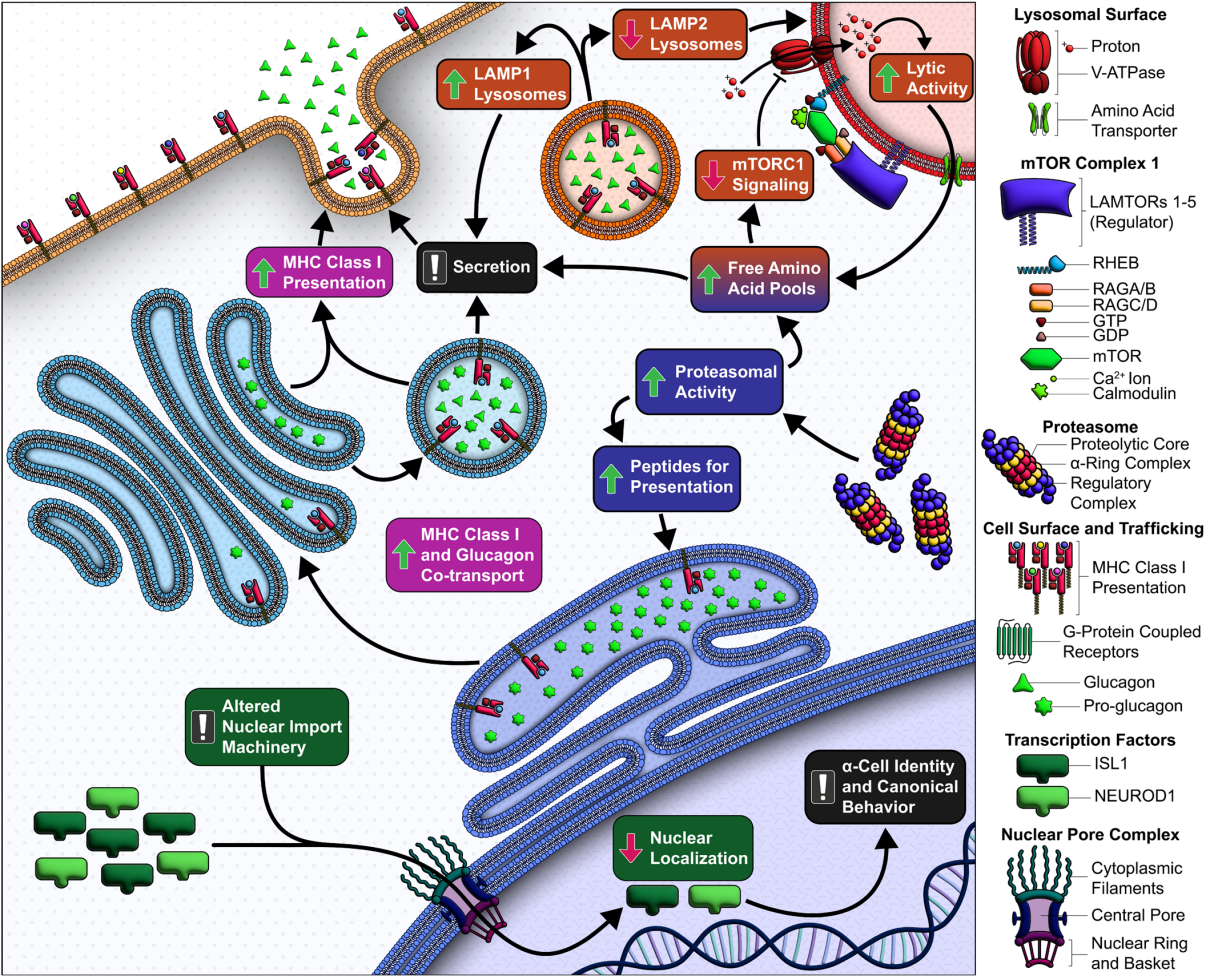
Summary of mechanisms contributing to α cell dysfunction in T1D. (Green) In T1D α cells, key transcription factors fail to localize to the nucleus, likely due to reduced nuclear import factor expression, impairing α cell gene regulation and identity. (Pink) MHC class I (MHC-I) molecules are aberrantly upregulated and colocalize with glucagon, suggesting that chronic cytokine exposure disrupts intracellular trafficking and secretory control. (Purple) Immune pressure is further supported by elevated (immuno)proteasome expression, promoting MHC-I peptide presentation. Increased proteasomal activity may also raise intracellular amino acids, stimulating glucagon release and activating mTORC1. (Orange) Despite this, mTORC1 components are downregulated in T1D α cells, disrupting lysosomal feedback. The resulting imbalance between LAMP1^+^ (secretory) and LAMP2^+^ (degradative) lysosomes likely impairs glucagon degradation and contributes to secretory dysregulation.

In summary, we identify molecular and functional differences in α and β cells from donors with T1D, consistent with disrupted glucagon and insulin secretion. A deeper analysis of α cells (Figure 8) revealed transcriptomic alterations linked to elevated immune signaling and antigen presentation, alongside impairments in mTOR signaling and lysosomal balance. Notably, increased proteasomal activity, associated with improved model scoring in both ND and T1D, could alter intracellular amino acid pools and contribute peptides for MHC-I loading and presentation. This is further complicated by defects in mTORC1 assembly, which may disrupt amino acid sensing and lysosomal homeostasis. Additionally, the colocalization of MHC-I with glucagon in T1D α cells suggests a breakdown in compartmentalization, potentially driven by immune signaling, causing MHC-I upregulation to the cell surface. While α cell lineage transcription factors such as NEUROD1 and ISL1 are upregulated in T1D α cells, their functional impact may be limited by reduced nuclear localization. Future studies could investigate variations in the nuclear localization sequences of these TFs in conjunction with importins (90) to better understand this exclusion. Finally, some of the transcriptional correlates of functional alterations in T1D α cells overlap with genetic risk loci, aligning with previous findings (22) that α cell dysfunction may even precede symptomatic onset of T1D. Overall, our findings highlight multiple candidate pathways and targets — several of which we validated — contributing to α cell dysfunction in T1D pathophysiology.

### Limitations.

We carefully selected control donors matched to the T1D cohort; however, both groups were skewed toward male donors due to limited donor availability. While this study links functional and molecular phenotypes of α cells in T1D, we acknowledge that dissociated islet cells may not fully reflect the behavior of cells in intact tissue. This discrepancy may arise from the loss of paracrine signaling, cell-cell interactions, and other structural features of the islet microenvironment. Ongoing studies using live pancreas slices may help address these limitations (9, 14, 74), although a direct connection between molecular profiles and in situ (dys)function remains a challenge. Although we observe clear phenotypic differences between cells from donors with and without diabetes, it remains possible that culture time could alter cellular transcriptomes and physiology, as some recovery of functionality has been suggested (12, 16). We performed validation studies using tissue sections and glucagon secretion measurements from intact islets, providing additional confidence that key single-cell findings are preserved in native tissue. Furthermore, our ability to detect canonical T1D pathways supports the validity of our results, indicating that, despite potential recovery effects from culturing, disease-relevant functional differences remained.

While Spearman correlations, which we previously used (16, 25), are well suited to detect monotonic relationships (i.e., those that continuously increase or decrease) without assuming a specific functional form, this approach may overlook nonmonotonic associations between gene expression and electrophysiological properties (like parabolic relationships). Future studies could apply alternative strategies, including generalized additive models, kernel-based dependence measures, or mutual information methods, to identify more complex patterns of association. However, these approaches are computationally more demanding and can be more sensitive to the inherent noise in biological data, especially with low-throughput patch-seq data. In all cases, experimental validation of key findings and hypotheses remains essential to substantiate the observed associations.

Ideally, protein-level validation would be performed using direct biochemical approaches such as immunoprecipitation or Western blotting. However, limited availability of T1D donor tissue and the low abundance of islets in the pancreas result in insufficient protein yield, particularly following cell-type sorting. Additionally, recapitulating the T1D phenotype in experimental systems remains challenging, as current animal models do not fully reflect the human condition (91). As a result, we relied on biopsy-based imaging to assess protein localization in situ, using only ratiometric or colocalization-based quantification methods that self normalize within each sample. We avoided direct comparisons of fluorescence intensity across samples, which are prone to variability due to factors such as FFPE tissue size, preservation time, sectioning variability, and other technical limitations beyond our control.

We observed altered MHC-I colocalization with glucagon and reduced nuclear accessibility in T1D α cells, likely linked to immune pathway upregulation, but did not examine the underlying mechanisms. This would require a detailed analysis of endolysosomal trafficking and nuclear pore composition. Whether enhancing nuclear permeability restores α cell secretory function in T1D remains unclear. Future studies could examine how inflammatory responses affect nuclear access and transcription factor activity. Although mTOR inhibition in ND islets increases glucagon secretion, it does not fully recapitulate the T1D α cell phenotype, where reduced expression of mTORC1 components likely impairs complex assembly. As such, it remains unclear whether mTOR agonism would restore behavior without first reestablishing complex integrity.

Finally, our findings primarily focus on α cells and longstanding disease, given that most donors were studied many years after T1D diagnosis. This, along with the generally low-throughput nature of the patch-seq approach, likely contributed to the low number of β cells collected. Thus, while patch-seq is a powerful method for studying islet cell physiology (16, 92) and diabetes (25), our β cell findings should be considered in the context of the limited number of T1D cells collected. Similarly, the α cell dysfunction studied here should be considered in the context of established T1D, although this remains quite relevant, given the continued importance of glucagon dysregulation. We suggest that studies on samples from donors with long-standing T1D highlight relevant aspects of the disease and are complementary to studies focused on autoantibody-positive or recent-onset donors (23, 93–95), which provide important insights into early disease stages.

## Methods

### Sex as a biological variable.

T1D samples were collected ‘as available’, and control samples were matched for sex (Supplemental Figure 3). Some α and β cell electrophysiological parameters appeared to be impacted by sex (Supplemental Figure 6). This was accounted for, in part, via our electrophysiological fingerprinting approach, which we showed was not impacted by donor sex in either the ND or T1D cohorts (Supplemental Figure 6).

### Human islets from donors with and without T1D.

Most human islets used in this study were isolated at the Alberta Diabetes Institute IsletCore using established protocols (96), with detailed donor information, quality control, and phenotyping assays available at www.HumanIslets.com (28). Additional T1D samples were obtained from nPOD (the Network for Pancreatic Organ Donation), HPAP (Human Pancreas Analysis Program) (97), and Rita Bottino (Imagine Islet Center - Imagine Pharma). Donor details are provided in Supplemental Table 1. T1D status was verified using reported diagnosis, pancreas weight, insulin content, and insulin immunostaining. When needed, further validation included autoantibody testing and MODY mutation screening. Human islets and dispersed cells were cultured in low glucose (5.5 mM) DMEM (Thermo-Fisher, #11885) with 10% FBS (Thermo-Fisher, #12483020) and 100 U/ml penicillin/streptomycin (Thermo-Fisher, #15070063) at 37°C and 5% CO_2_ for 1–3 days prior to use. The T1D cohort included all patch-seq α and β cells from diagnosed donors (ages 13–44 years; BMI 17.0–29.4 kg/m²; CIT 2–24.5 h). Control (ND) patch-seq cells were selected from donors without diabetes and with comparable age, BMI, and CIT ranges (12–46 years; 16.4–27.5 kg/m²; 2–23 h). ND donors outside these ranges were excluded to minimize confounding effects.

### Hormone secretion assays.

Dynamic glucagon and insulin secretion were measured using 35 islets per condition, preincubated for 30–45 min in KRB buffer containing (in mM): 140 NaCl, 3.6 KCl, 2.6 CaCl_2_, 0.5 NaH_2_PO_4_, 0.5 MgSO_4_, 5 HEPES, 2 NaHCO_3_, 0.5 mg/ml essentially fatty acid-free BSA, and glucose as indicated. Perifusion was performed using the Biorep Peri4 system with KRB containing glucose, GIP (Anaspec), alanine (Sigma), and mTOR inhibitor Torin-2 (MedchemExpress, #HY-13002), as noted in figure legends. Total hormone content was assessed by lysing islets in acid ethanol. All samples were treated with 5 μg/ml aprotinin (Sigma) to prevent degradation and stored at –20 °C for ELISA analysis of glucagon (MSD; K1515YK-2) and insulin (Alpco; 80-INSHU-CH01).

### Electrophysiology.

K_ATP_ channel activity was recorded in whole-cell configuration upon washout of intracellular ATP. Currents were recorded in response to voltage steps from −60 and −80 mV, holding at −70 mV. The bath solution contained (in mM): 138 NaCl, 5.6 KCl, 1.2 MgCl_2_, 2.6 CaCl_2_, 5 HEPES, and 5 glucose (pH 7.4). The pipette solution contained: 125 KCl, 30 KOH, 1 MgCl_2_, 10 EGTA, 5 HEPES, 0.3 Mg-ATP, and 0.3 K-ADP (pH 7.15).

Action potentials were recorded in current-clamp mode via perforated patch-clamp. The bath solution contained (in mM): 140 NaCl, 3.6 KCl, 1.5 CaCl_2_, 0.5 MgSO_4_, 10 HEPES, 0.5 NaH_2_PO_4_, 5 NaHCO_3_, and glucose as indicated (pH 7.3 with NaOH). Patch pipettes were filled with (in mM): 76 K_2_SO_4_, 10 KCl, 10 NaCl, 1 MgCl_2_ and 5 HEPES (pH 7.25 with KOH), and backfilled with 0.24 mg/ml amphotericin B (Sigma, #A9528). Seal stability (> 10 GΩ) and access resistance were monitored for quality control.

Cell identity was confirmed by immunostaining (1:200) for glucagon (Sigma, #G2654) and insulin (DAKO/Agilent, #IR00261-2), visualized (1:200) with Alexa Fluor 594 goat anti-mouse (Thermo-Fisher, #A-11032) and Alex Fluor 488 (Thermo-Fisher, #A-11073), respectively. Data were analyzed using FitMaster (HEKA Electronics) and Prism 10 (GraphPad, San Diego, CA).

Electrophysiology for patch-seq was performed as described previously (16, 25). Briefly, hand-picked islets were dissociated into single cells using StemPro accutase (Thermo-Fisher, #A11105-01) and plated on 35-mm dishes for patch-clamp. Cells were cultured in low-glucose (5.5 mmol/L) DMEM with L-glutamine, 110 mg/L sodium pyruvate, 10% FBS, and 100 U/mL penicillin/streptomycin for 1–3 days before recordings. Whole-cell and perforated patch-clamp were performed in a heated bath (32–35^o^C) using fire-polished pipettes (4–5 MΩ). Exocytosis was measured by whole-cell capacitance responses using the Sine+DC lock-in function of a HEKA EPC10 amplifier and PatchMaster software (HEKA Electronics, Germany). Cells were depolarized 10 times for 500 ms to 0 mV from a –70mV holding potential. Ca^2+^ and Na^+^ currents were evoked by membrane potentials ranging from –70 to –10mV. Responses were recorded 1–2 mins after establishing whole-cell configuration. The bath solution contained (in mM): 118 NaCl, 20 tetraethylammonium-Cl, 5.6 KCl, 1.2 MgCl_2_, 2.6 CaCl_2_, 5 HEPES, and 5 glucose (pH 7.4 with NaOH). Patch pipettes were filled with (in mM): 125 Cs-glutamate, 10 CsCl, 10 NaCl, 1 MgCl_2_, 0.05 EGTA, 5 HEPES, 0.1 cAMP and 3 MgATP (pH 7.15 with CsOH).

### Single-cell RNA-seq and analysis.

Sequencing was performed on patched cells collected via a separate wide-bore pipette (0.2–0.5 MΩ) containing lysis buffer (10% Triton, Sigma-Aldrich, #93443; RNase inhibitor 1:40, Clontech, #2313A), ERCC RNA spike-in mix (1:600,000; Thermo-Fisher, #4456740), 10 mM dNTP, and 100 μM dT primer (3’-AAGCAGTGGTATCAACGCAGAGTACTTTTTTTTTTTTTTTTTTTTTTTTTTTTTTVN-5′). Samples were stored at –80°C in PCR tubes. cDNA and sequencing libraries were generated using an adapted SmartSeq-2 protocol optimized for patch-seq (16, 25). Libraries were prepared by cDNA amplification via Tn5 tagmentation and sequenced on an Illumina NovaSeq with paired-end 100 bp reads, achieving approximately 1 million reads per cell. Reads were aligned to the human genome (GRCh38 with ERCC sequences) using STAR (98), with gene counts quantified by htseq-count (intersection-nonempty) using Ensemble89 GTF annotations (99).

Cell typing of patch-seq cells was performed in ScanPy using all ND and T1D patch-seq cells. Gene expression was normalized to 10,000 counts and log transformed. Total counts and mitochondrial percentage were regressed out prior to dimensionality reduction and Leiden clustering. Clusters expressing insulin (*INS*) and islet amyloid polypeptide (*IAPP*) were identified as β cells; glucagon (*GCG*) as α cells; somatostatin (*SST*) as δ cells: pancreatic polypeptide (*PPY*) as γ cells; serine protease 1 and 2 (*PRSS1, PRSS*2) as acinar cells; and ghrelin (*GHRL*) as ε cells (undetected).

Differential expression analysis (DEA) was performed using the Wilcoxon rank-sum test to compare transcriptomes of α and surviving β cells from T1D and matched ND donors. Total counts were normalized to 1,000,000 for consistency with previous studies (16, 25). After UMAP generation, overlaid metadata included donor ID, cell type, HbA1c, diabetes status, BMI, years with diabetes, age, sex, and electrophysiological features (cell size, total exocytosis, sodium currents). To identify enriched pathways, Gene Set Enrichment Analyses (GSEA) was performed using WebGestalt (100) on DEA scores, applying a false discovery rate (FDR) cutoff of 0.1, and weighted set cover to reduce redundancy.

### Patch-seq analysis.

Electrophysiological fingerprinting was performed using a gradient-boosted decision tree ensemble as previously described (25). Specifically, we used Extreme Gradient Boosting (XGBoost v1.6.2) in Python (v3.7.11) to classify cells as α or β cells based solely on electrophysiological data — without prior knowledge of cell identity — and to assign a confidence score. The model was trained on data from the ND patch-seq cohort and immunostained ND cells matched by age, BMI, and CIT (α cells: 248+9; β cells: 104+186). Features included: cell size (pF), normalized total, first and late depolarization capacitances (fF/pF), early and late peak calcium current amplitudes (pA/pF), calcium integral (pC/pF), and peak sodium current (pA/pF). These were chosen to provide balanced insight into cell size, excitability (ion currents), and secretory activity (exocytosis) with reasonable throughput, extending previously published work (25, 27). Hyperparameter tuning targeted ≥ 75% accuracy, with early stopping (100 iterations) and AUCPR as the evaluation metric. Model performance was assessed using confusion matrices (scikit-learn v1.0.2), and feature importance plots confirmed appropriate use of all parameters without overreliance on any single feature (16). When applied to ND and T1D patch-seq cells, model scores were interpreted as α probability: 1.0 indicating α-like, 0.0 β-like electrophysiological profiles. Associations between model scores, electrophysiological features, and donor characteristics were tested via Ordinary Least Squares (OLS) regression (statsmodels v0.12.2; scikit-learn v1.0.2).

Spearman’s rank correlations were computed using SciPy between transcript expression and electrophysiological features, only including transcripts detected in ≥ 50% of cells to ensure populational relevance. Then, for each gene, cells with undetectable expression (0 counts) were excluded from that specific correlation to minimize the influence of tied ranks and zero inflation. Bootstrapping (1,000 iterations) was used to generate mean correlation coefficients for gene set enrichment analysis (GSEA).

α cell protein-protein interaction (PPI) networks were generated using Metascape (101). Genes with significant differential expression in ND or T1D α cells (|log_2_FC| > 1, p < 0.05) were submitted for pathway enrichment and PPI analysis using the MCODE algorithm. Only nodes with ≥ 3 connections are shown.

Enrichment of genes associated with T1D risk alleles was performed by compiling gene sets from multiple sources. We used the GeneSifter tool on the T1D Knowledge Portal (102) (T1DKP; https://t1d.hugeamp.org/) to identify genes with TPM ≥ 1 in pancreas and a T1D Human Genetic Evidence (HuGE) score ≥ 3 (103). Islet expression QTL (eQTL) data was from the InsPIRE study (104) for variants associated with T1D in a published GWAS (89) (*P* < 5 × 10^-8^). Strict and permissive eQTL sets were defined using significance thresholds of 2 × 10^-6^ and 5 × 10^-^³, respectively. We used Fisher’s Exact Test to assess enrichment of T1D-associated genes in those enriched in ND and T1D α cells, comparing observed overlaps to the background of all tested genes and calculating 2-sided *P* values.

### Pancreatic biopsy imaging.

Formalin-fixed paraffin-embedded (FFPE) pancreas biopsies were sectioned at 4 μm by the Alberta Diabetes Institute HistoCore. Slides were baked at 58°C for 20 min, then deparaffinized and rehydrated using sequential xylene, ethanol, and water washes. Antigen retrieval was performed using citrate buffer (10mM citrate, 0.05% Tween-20, pH 6.0) by microwave (680 W, 16 min). Each subsequent step included 3 PBS washes. Permeabilization used 0.1% Triton X-100 (10 min) and 20% goat serum blocking (30 min, room temperature). Primary antibodies (except antiglucagon) were diluted in 4% goat serum (1–2 μg/mL), applied as 175 μL droplets, and incubated overnight at 4°C. Secondary antibodies were diluted to 5 μg/mL in 4% goat serum and incubated for 1 hr at room temperature. Anti-glucagon antibody was applied (1 μg/mL, 30 min, room temperature), followed by its secondary and DAPI (20 min). Mounting used 50 μL ProLong Glass (Thermo-Fisher, P36984) with 170 μm high-precision #1.5H coverslips (Fisher-Scientific, #NC1776158) and cured overnight at room temperature. Primary antibodies used against: Glucagon (Thermo-Fisher, #A-11029), HLA-Class I ABC (Abcam, #ab70328), ISL1 (Thermo-Fisher, # PA527789), NEUROD1 (Abcam, #ab60704), LAMP1 (Thermo-Fisher, #21997-1-AP), LAMP2 (Thermo-Fisher, #66301-1-IG). Secondary antibodies used: Anti-mouse Alexa Fluor 488 (Thermo-Fisher, #A-11029), anti-rabbit Alexa Fluor 568 (Thermo-Fisher, #A-11036), anti-guinea pig Alex Fluor 647 (Thermo-Fisher, #A-21450).

Images were acquired on a Leica STELLARIS-DMI8 with a 63x oil-immersion lens (NA 1.4, RI 1.518). Excitation was performed through the LAS X (v5.2.2) dye-assistant with emission gating to eliminate crosstalk. Detection was 8-bit photon counting, and excitation parameters kept constant within experiments. Multi-channel 3D Z-stacks were acquired at 1024x1024 resolution with 0.2μm steps. All channels were acquired prior to proceeding to the next plane. LAS X Lightning performed deconvolution

Image analysis was performed in Fiji (v1.54k.). Lightning deconvolution heatmaps of the glucagon signal were used to define α cell boundaries for quantifying signals within glucagon-positive areas. Ratiometric quantification was performed on raw “summation” hyperstacks. Nuclear signal (%) was measured using user-defined ROIs based on DAPI and glucagon signals. 3D colocalization analyses were performed on deconvoluted images using Fiji’s Coloc2 plugin.

### Figure generation.

Transcriptomic figures were generated using Scanpy (v1.9.3), Seaborn (v0.12.2), and Matplotlib (v3.4.2). Other plots were created in GraphPad Prism 10. Microscopy images were exported by Fiji (v1.54k) Figure layouts were assembled in Adobe Illustrator (v28.5).

### Statistics.

*P* values under 0.05 and false discovery rates under 0.1 were considered significant, unless otherwise stated. Sample sizes, statistical methods, algorithms, and software are described in the figure legends and Methods and include Mann-Whitney, 2-way ANOVA, unpaired 2-tailed *t* tests, Fisher’s exact test, Kruskal-Wallis with Dunn’s correction, and Spearman’s rank correlations. Box-and-whisker plots are 10th–90th percentile, and error bars are standard deviation unless stated otherwise in the figure caption.

### Study approval.

All procedures were approved by the Human Research Ethics Board at the University of Alberta (Pro00013094; Pro00001754), with informed consent obtained from donor families.

### Data availability.

Raw sequencing data are available via NCBI GEO and SRA under accession numbers GSE124742, GSE164875, and GSE270484. Patch-seq and related human islet data can be accessed at HumanIslets.com.

## Author contributions

PEM and SRQ conceived the study. TDS performed experimental designs and interpretations. XQD performed electrophysiology. JCS, RCJ, NFN, AMD, and MT performed sequencing. TDS analyzed RNA-seq with JDE, CEE, JCS, and JX. HMM and KJG performed GWAS analyses. Electrophysiological fingerprinting was performed by TDS and PEM, with contributions from XQD, AB, and JCS. AFS and TDS performed perifusion experiments. Islet isolation and biopsies were conducted by JGL, NS, AB, and JEMF. Microscopy was performed by TDS. Protein-protein network analysis was performed by RAeD. Figures and manuscript were composed by TDS and PEM. All authors contributed manuscript editing. PEM is the guarantor of this work and responsible for data access.

## Funding support

This work is the result of NIH funding, in whole or in part, and is subject to the NIH Public Access Policy. Through acceptance of this federal funding, the NIH has been given a right to make the work publicly available in PubMed Central.

- The Canadian Institutes of Health Research (MacDonald: 186226).
- Breakthrough T1D (MacDonald: 2-SRA-2019-698-S-B).
- The National Institutes of Health (MacDonald: DK120447; Gaulton: DK105554, DK138512, HG012059).
- HPAP (RRID: SCR_016202), a Human Islet Research Network (RRID: SCR_014393) consortium (UC4-DK-112217, U01-DK-123594, UC4-DK-112232, and U01-DK-123716).
- The Alberta-Helmholtz Diabetes Research School (TDS).
- The Alberta Innovates Scholarship in Data-Enabled Innovation (TDS).
- The Sir Fredrik Banting and Dr. Charles Best Canada Graduate Scholarship-(MSc.) (TDS).
- The DT O’Connor Scholar in Genetics award (UC San Diego) (HMM).
- The Knut and Alice Wallenberg Foundation (Wallenberg Molecular Medicine Fellow) (JCS).
- The Swedish Research Council (grant 2021-05109) (JCS).
- The Erling Persson Foundation (Swedish Foundations’ Starting Grant) (JCS).
- Endowed chair postion UC San Diego (KJG).
- Canada Research Chair in Islet Biology (PEM).

## Supplementary Material

Supplemental data

Supplemental table 1

Supplemental table 2

Supplemental table 3

Supplemental table 4

Supplemental table 5

Supporting data values

## Version 1 09/30/2025

In-Press Preview

## Version 2 12/01/2025

Electronic publication
