# Supplementary material for: Altered immune and metabolic molecular pathways drive islet cell dysfunction in human type 1 diabetes
Source: J Clin Invest. 2025 Sep 30;135(23):e195267. doi: 10.1172/JCI195267 (PMC12646676; doi:10.1172/JCI195267)

SUPPLEMENTARY MATERIALS

Altered immune and metabolic molecular pathways drive islet cell dysfunction in human type 1 diabetes

Theodore dos Santos[1,2], Xiao Qing Dai[1,2], Robert C Jones[3,4,5], Aliya F Spigelman[1,2], Hannah M Mummey[6], Jessica D Ewald[7], Cara E Ellis[1,2], James G Lyon[2], Nancy Smith[1,2], Austin Bautista[2], Jocelyn E Manning Fox[1,2], Norm F. Neff[4], Angela Detweiler[4], Michelle Tan[4], Rafael Arrojo E Drigo[8,9], Jianguo Xia[10], Joan Camunas-Soler[11,12], Kyle J Gaulton[13], Stephen Quake[3,4,5], Patrick E MacDonald[1,2,14]*

[1]Department of Pharmacology, University of Alberta, Edmonton, AB, Canada

[2]Alberta Diabetes Institute, University of Alberta, Edmonton, AB, Canada

[3]Department of Bioengineering, Stanford University, Stanford, CA , USA

[4]Chan Zuckerberg Biohub, San Francisco, CA, USA

[5]Department of Applied Physics, Stanford University, Stanford, CA, USA

[6]Bioinformatics and Systems Biology Program, University of California San Diego, La Jolla CA

[7]European Bioinformatics Institute (EMBL-EBI), European Molecular Biology Laboratory, Wellcome Genome Campus, Hinxton, UK

[8]Department of Molecular Physiology and Biophysics, Vanderbilt University, Nashville, TN, USA

[9]Center for Computational Systems Biology, Vanderbilt University, Nashville, TN, USA

[10]Department of Microbiology & Immunology, McGill University, Montreal, QC, Canada

[11]Department of Medical Biochemistry and Cell Biology, Institute of Biomedicine, University of Gothenburg, Sweden

[12]Wallenberg Centre for Molecular and Translational Medicine, Sahlgrenska Academy, University of Gothenburg, Sweden

[13]Department of Pediatrics, Pediatric Diabetes Research Center, University of California San Diego, La Jolla CA, USA

[14]Division of Metabolism, Endocrinology & Diabetes, University of Michigan, Ann Arbor, MI, USA

Supplementary Table 1

1.1. Summary of donors, their characteristics, and use of their tissue in the experiments and analyses performed in this study.

1.2. T1D Donors subjected to the patch-seq method

Supplementary Table 2

2.1. Differential expression analysis

2.2. Pathway analysis in alpha cells, with overlapping pathways minimized by weighted set coverage

2.3. Pathway analysis in alpha cells, including all pathways

2.4. Pathway analysis in beta cells, with overlapping pathways minimized by weighted set coverage

2.5. Pathway analysis in beta cells, including all pathways

Supplementary Table 3

3.1. Correlations between cell modelling scores and transcript expression in T1D alpha cells

3.2. Correlations between cell modelling scores and transcript expression in ND alpha cells

3.3. Pathway analysis of T1D and ND alpha cell transcript correlates to total exocytosis. Showing all pathways, or with overlapping pathways reduced by weighted set cover

3.4. Pathway analysis of T1D and ND alpha cell transcript correlates to model scoring. Showing all pathways, or with overlapping pathways reduced by weighted set cover

Supplementary Table 4

4.1. Pathway analysis of transcripts that correlate with a 'pro-secretory' phenotype in T1D alpha cells, shown as all pathways or with overlapping pathways reduced by weighted set cover

4.2. Pathway analysis of transcripts that correlate with a 'anti-secretory' phenotype in T1D alpha cells, shown as all pathways or with overlapping pathways reduced by weighted set cover

4.3. Pathway analysis of transcripts that correlate with a 'pro-secretory' phenotype in ND alpha cells, shown as all pathways or with overlapping pathways reduced by weighted set cover

4.4. Pathway analysis of transcripts that correlate with a 'anti-secretory' phenotype in ND alpha cells, shown as all pathways or with overlapping pathways reduced by weighted set cover

Supplementary Table 5

5. Integrated differential transcript expression and correlation to cell behaviour

Supplemental Figure 1: Cell typing of ND and T1D patch-seq data

(A) Uniform manifold approximation and projection (UMAP) of ND (n=1435, 46 donors) and T1D (n=990, 10 donors) patch-seq cells (left). Leiden clustering revealed 15 groups, represented by the UMAP colours. The expression analysis represented as a dot plot (right) indicated the cell type in each cluster based on the expression of islet cell type markers. Alpha cell clusters (A1 to A11) were identified by the expression of glucagon (GCG), beta cells (B1) identified by insulin (INS) and islet amyloid polypeptide (IAPP), delta cells (G1) by somatostatin (SST), gamma cells (G1) by pancreatic polypeptide (PPY), and acinar cells (Ac) by serine protease 1 (PRSS1) and 2 (PRSS2). Ghrelin (GHRL) expression, indicative of epsilon cells, was not prominently detected.

(B) UMAP from A with islet cell type marker expression overlays, indicating the identification of cell types. The colour bar represents the indicated marker's expression (Log_2[Counts per million +1]).

(C) Post-cell typing, clusters of similar cell types were merged to identify all the alpha, beta, gamma, delta, and acinar cells in the patch-seq data, represented as the colours in the UMAP (left). Expression levels of islet identity markers post-merge are shown as a dot plot (right). The colour bar represents the mean expression in the group, while the size of the dot indicates the proportion of cells in the group expressing the indicated transcript.

Supplemental Figure 2: UMAP of cell-typed patch-seq cells with donor metadata overlays

Post-cell typing, donor information and characteristics were overlaid onto the uniform manifold approximation and projection (UMAP) shown in supplementary figure 1A. Included are the age of the donor (years), body mass index (kg/m^2; BMI), glycated hemoglobin A1c (%; HbA1c), sex, diabetes status, duration of diabetes (years), and donor identification codes (ID) from the University of Alberta Islet Core, Network for Pancreatic Organ Donors with Diabetes (nPOD), and the Human Pancreas Analysis Program (HPAP).

Supplemental Figure 3: Comparison between the T1D and matched ND donors from which patch-seq data was analyzed

Comparison of age (years), body mass index (kg/m^2), cold ischemia time (hours), glycated hemoglobin A1c (%) and sex distribution of type 1 diabetes donors and matched non-diabetes control donors from which the patch-seq data was generated.

****$p < 0.0001$ as indicated using the two tailed unpaired t-test.

Supplemental Figure 4: UMAP of patch-seq data from T1D and matched control ND donors.

(A) Uniform manifold approximation and projection (UMAP) of T1D (n=692, 9 donors) and control ND (n=375, 17 donors) patch-seq cells of donors included for further analysis, with overlays of identified cell type (left), and a dot plot of expression for islet cell type markers (right) including glucagon (GCG) for alpha cells, insulin (INS) and islet amyloid polypeptide (IAPP) for beta cells, somatostatin (SST) for delta cells, pancreatic polypeptide (PPY) for gamma cells, and serine protease 1 (PRSS1) and 2 (PRSS2) for acinar cells.

(B) UMAP from A with islet cell type marker expression overlays, indicating the identification of cell types. The colour bar represents the indicated marker's expression (Log_2[Counts per million +1]).

(C) UMAP from A with donor diabetes status overlay for each cell (left), and a dot plot of their expression for islet cell type markers. The colour bar represents the mean expression in the group, while the size of the dot indicates the proportion of cells in the group expressing the indicated transcript.

Supplemental Figure 5: UMAP of patch-seq data from T1D and matched control ND donors with donor characteristics and electrophysiology

Donor information and characteristics were overlaid onto the uniform manifold approximation and projection (UMAP) shown in supplementary figure 4A. Included are the donor identification codes (ID) from the University of Alberta Islet Core, Network for Pancreatic Organ Donors with Diabetes (nPOD), and the Human Pancreas Analysis Program (HPAP), the age of the donor (years), body mass index (kg/m^2; BMI), glycated hemoglobin A1c (%; HbA1c), sex, diabetes status, duration of diabetes (years), and electrophysiology measures of cell size (pF), total exocytosis (fF/pF), and peak sodium (Na^+) current (pA/pF).

Supplemental Figure 6: Impact of donor characteristics on electrophysiology and α-score

Ordinary least squares (OLS) regression to identify trends between indicated electrophysiology or model score values against donor characteristics. The colour bar represents the significance of the regression. The OLS analysis was repeated for the T1D data with diabetes duration as an additional variable, which is not applicable for the ND data. Unlike the electrophysiology measures which show potential trends when compared against donor characteristics, the regression of model scoring appears largely unaffected by donor effects in the cohorts.

Supplemental Figure 7: Cell typing of ND and T1D patch-seq data

(A) Comparison of the electrophysiology and model scoring at differing glucose concentrations obtained from patch-seq alpha (α)-cells during whole-cell patch-clamp. At 1mM glucose, ND (13 donors) and T1D (4 donors) n-values for cell size = 156, 81; total exocytosis = 154, 81; early exocytosis = 154, 80; late exocytosis = 157, 80; peak Na^+ current = 154, 78; early Ca^{2+} current = 156, 80; late Ca^{2+} current = 154,80; Ca^{2+} charge entry = 149, 75; exocytosis normalized to Ca^{2+} = 136,74. At 10mM glucose, ND (10 donors) and T1D (4 donors) n-values for cell size = 85, 80; total exocytosis = 84, 80; early exocytosis = 84, 80; late exocytosis = 84, 80; peak Na^+ current = 84, 81; early Ca^{2+} current = 84, 80; late Ca^{2+} current = 83, 79; Ca^{2+} charge entry = 77, 70; exocytosis normalized to Ca^{2+} = 68, 69.

(B) Model scoring of α-cell (α-score) electrophysiology recorded at 5mM, 1mM, and 10mM from patch-seq α-cells of ND matched controls (17, 13, 10 donors respectively) and T1D (9, 4, 4 donors respectively). ND and T1D n-values for score at 5mM = 248, 596; 1mM = 158 ,82; 10mM = 85, 81.

(C) Cumulative frequency graph of α-scores of patch-seq α-cells patched at 1mM, 5mM, and 10mM glucose, showing differences in the proportion of cells and their respective scoring across glucose concentration, stratified for diabetes status.

*p < 0.05, **p < 0.01, ***p < 0.001, and ****p < 0.0001 as indicated using the two-tailed non-parametric Mann-Whitney test in (A) and using the non-parametric Kruskal-Wallis test with Dunn's correction (B). Outliers were determined based on |z-score| >3 and excluded from comparison and statistical analysis (A and B).

Supplemental Figure 8: Integrated analysis of notable transcript hits from pathways that may contribute to α-cell dysfunction

Bubble plot of notable transcript hits, combining their differential expression data, and correlations of expression with either total exocytosis or model score (α-score) in α-cells patch-clamped at 5mM glucose, stratified for diabetes status. Transcript names flanked by asterisks indicate significant (p<0.05) differential expression, with the colour indicating their

expression level in the cohort. Horizontal displacement of the bubble represents the correlation coefficient, while the size represents the significance of the correlation. Transcripts are grouped based on their shared physiological functions, indicated by the vertical labelled brackets.

Supplemental Figure 9: Linear regression of nuclear ISL1 and NEUROD1 signals

Linear regression between the nuclear presence of ISL1 and NEUROD1 in non-diabetes (ND) and type 1 diabetes (T1D) alpha cells, obtained from confocal fluorescent imaging of formalin fixed paraffin embedded samples of biopsies from donors that the patch-seq data was generated from. While the regression appears similar in the ND and T1D alpha cells, the latter shows an overall shift toward 0 on both axes.

$****p < 0.0001$ as indicated

Supplemental Figure 1

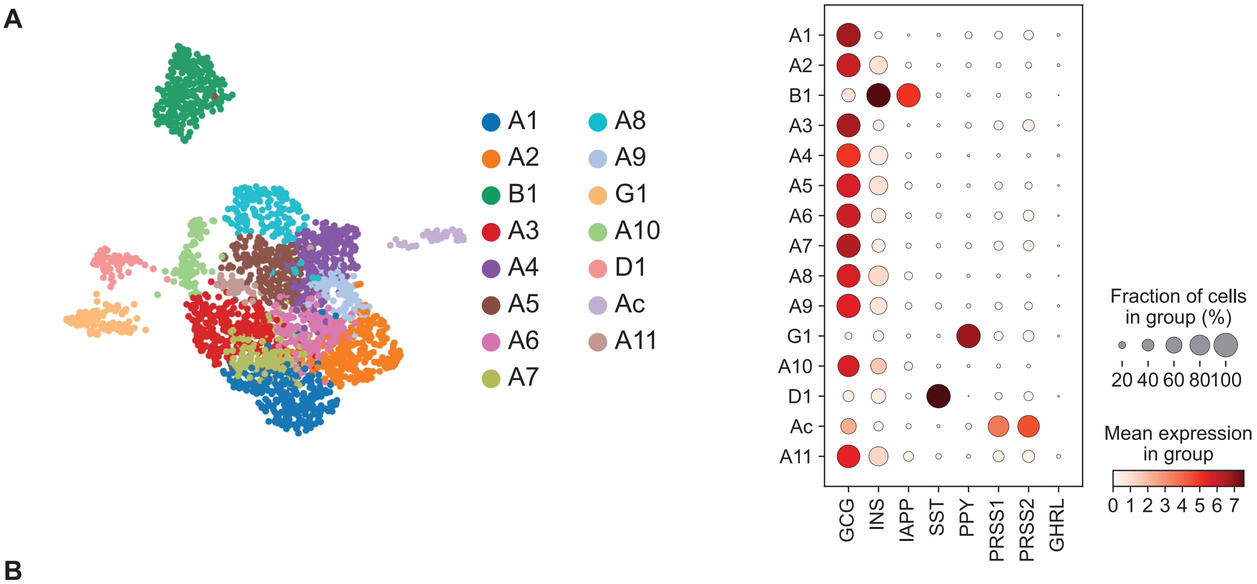

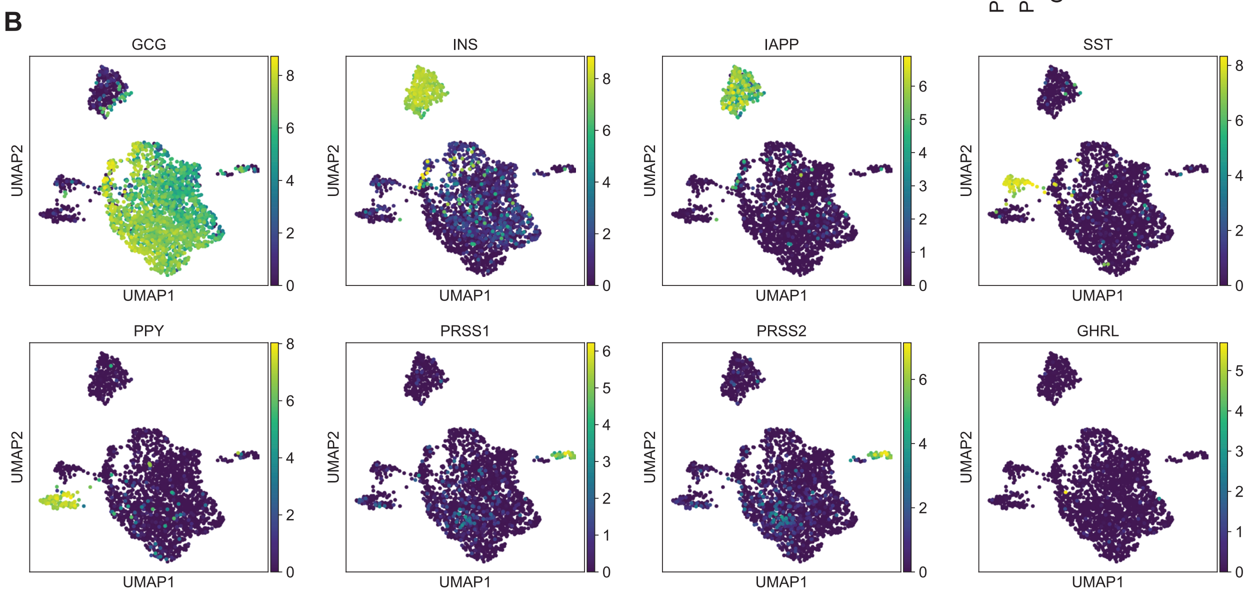

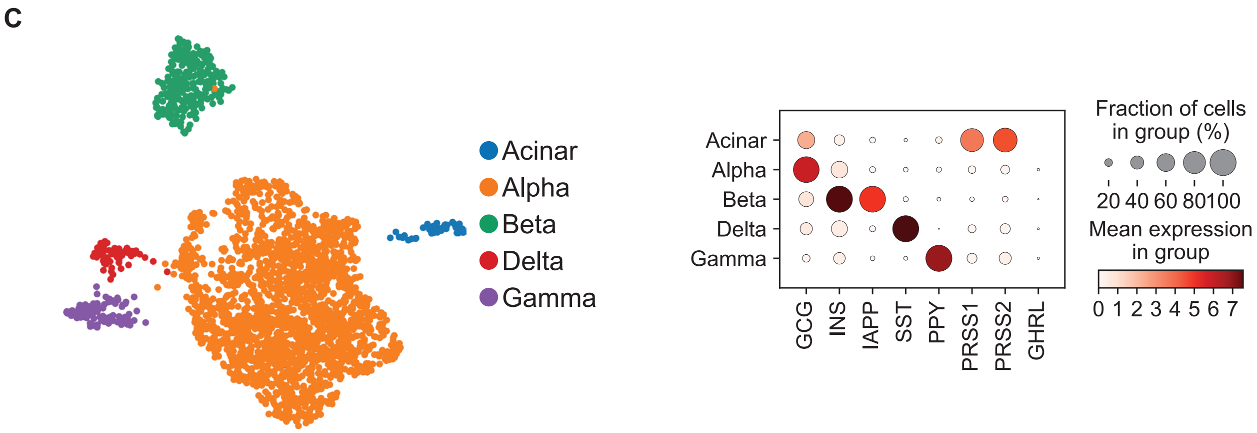

Supplemental Figure 2

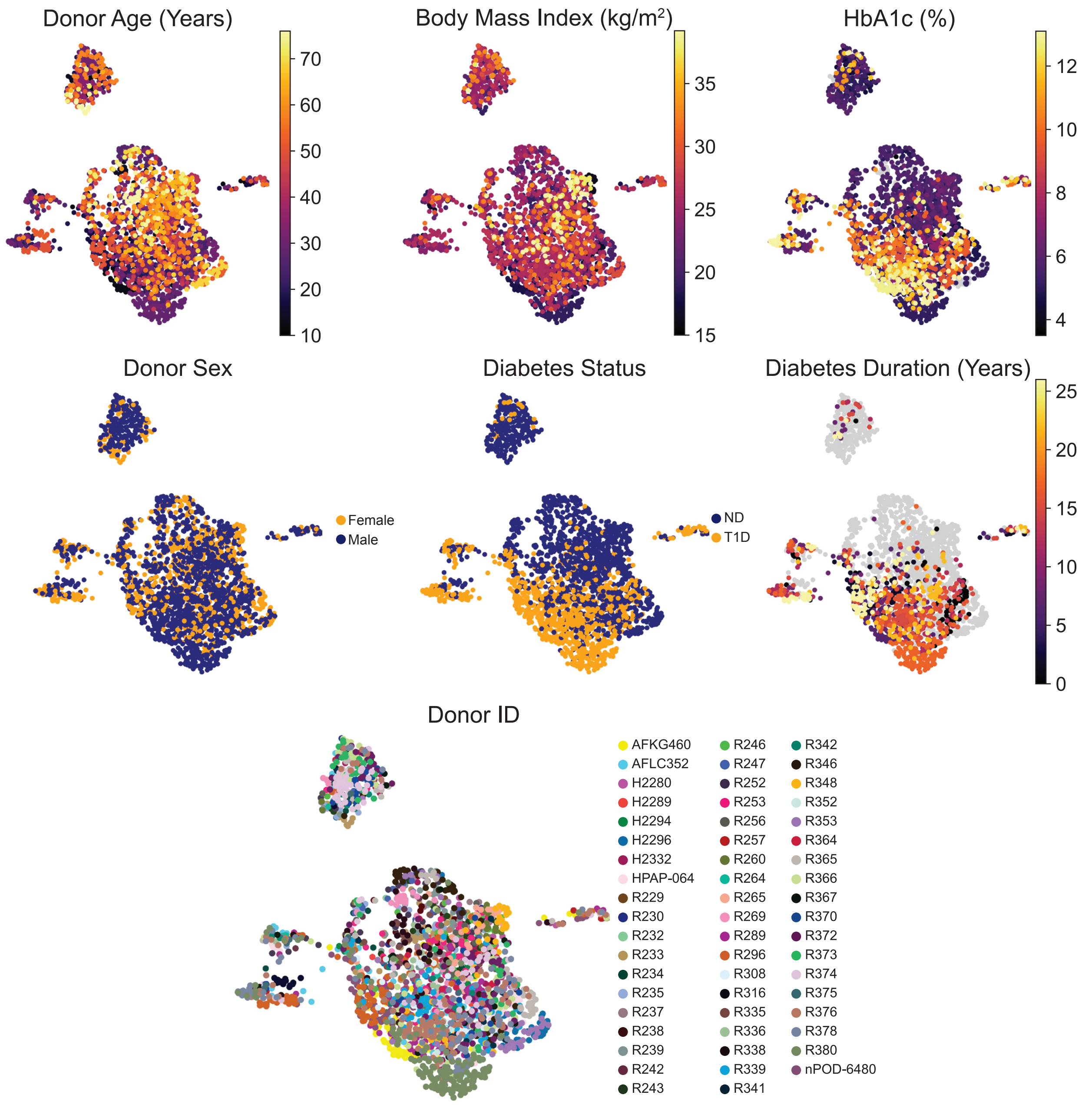

Supplemental Figure 3

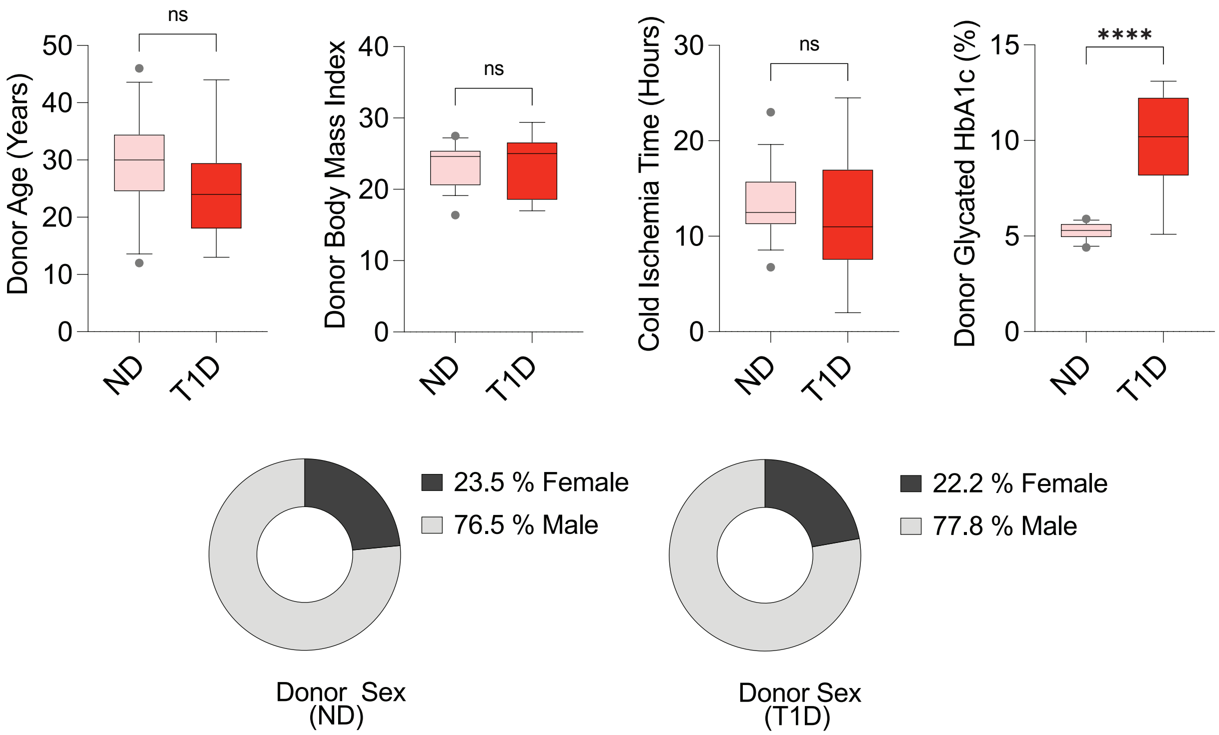

Supplemental Figure 4

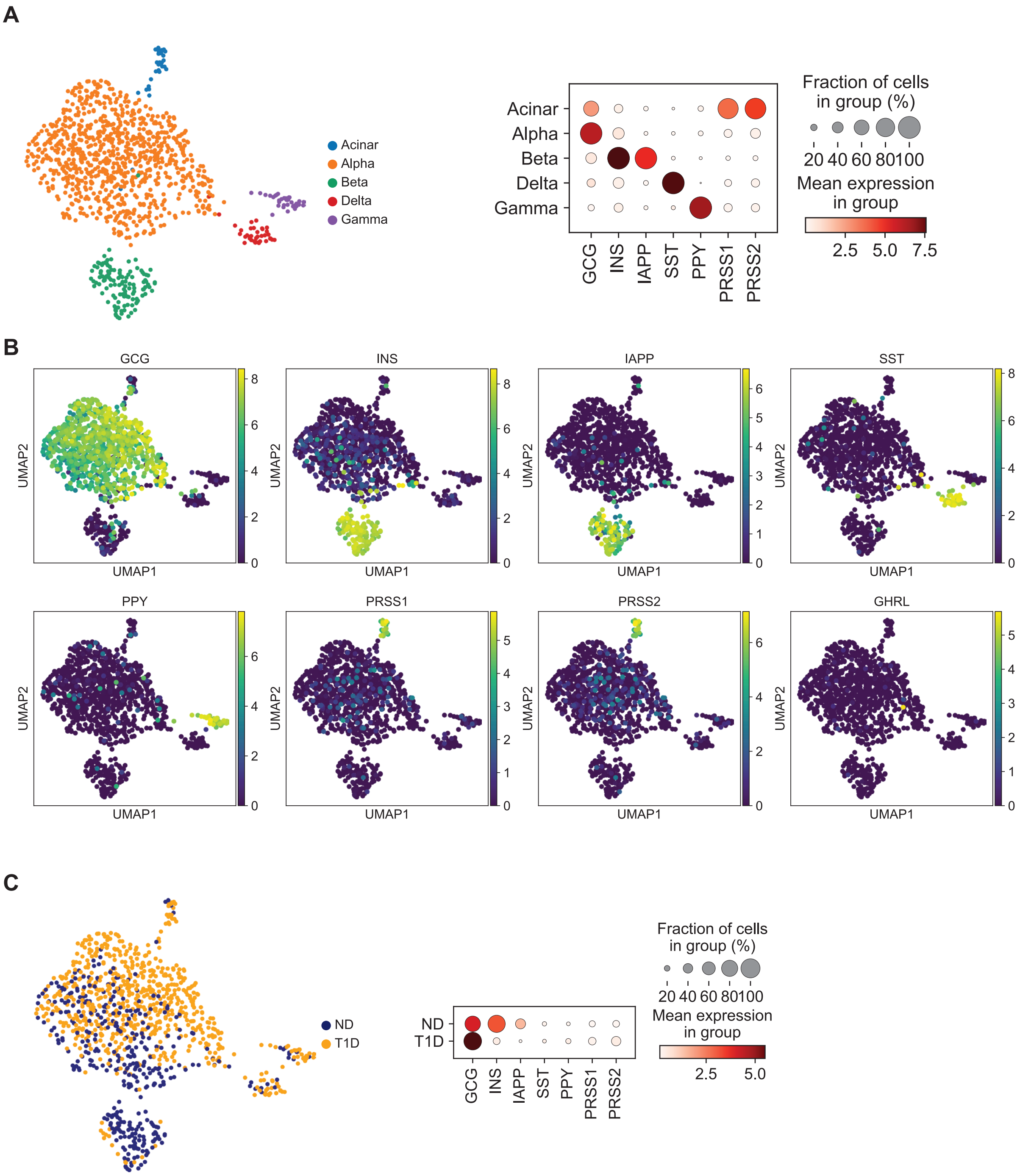

Supplemental Figure 5

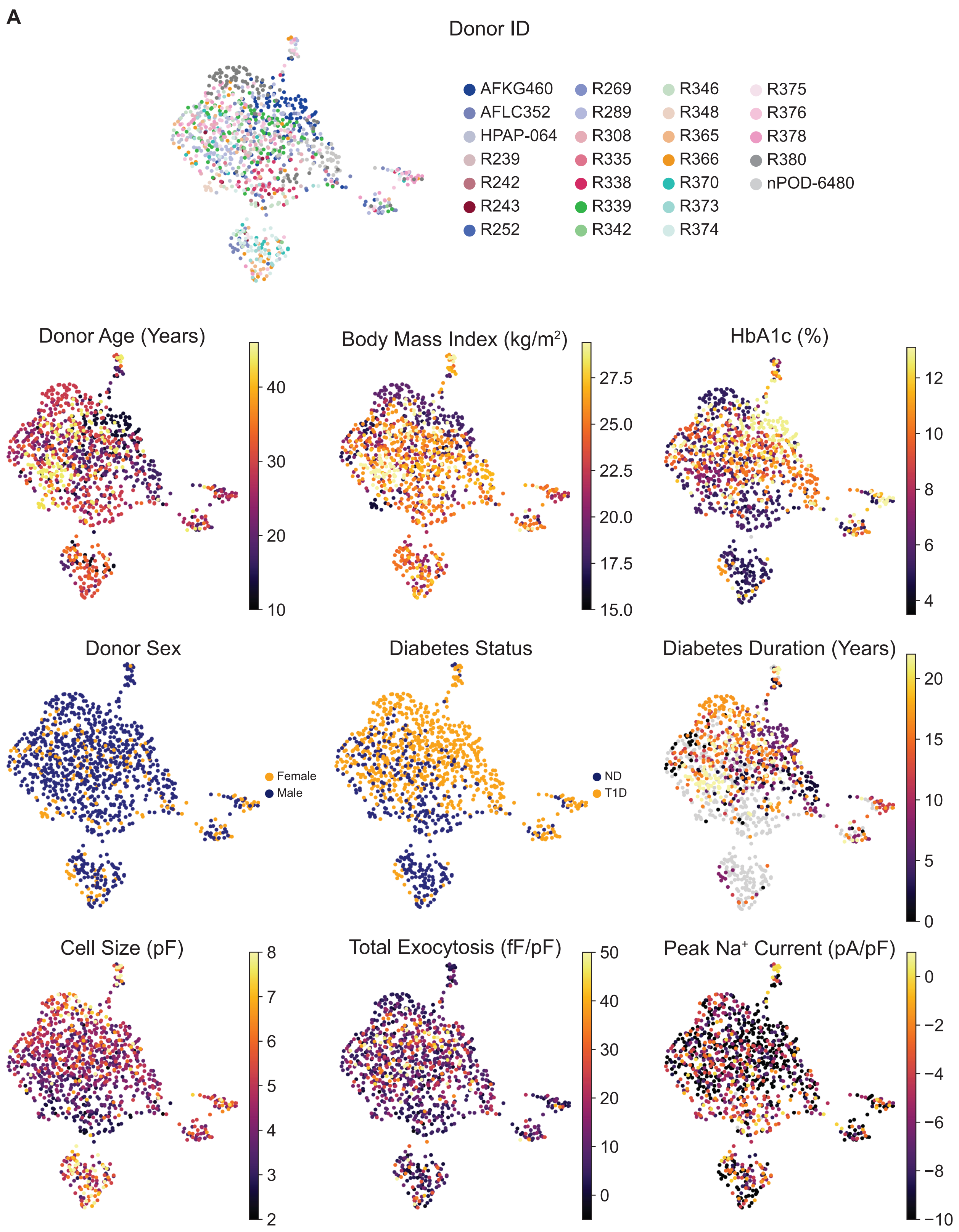

Supplemental Figure 6

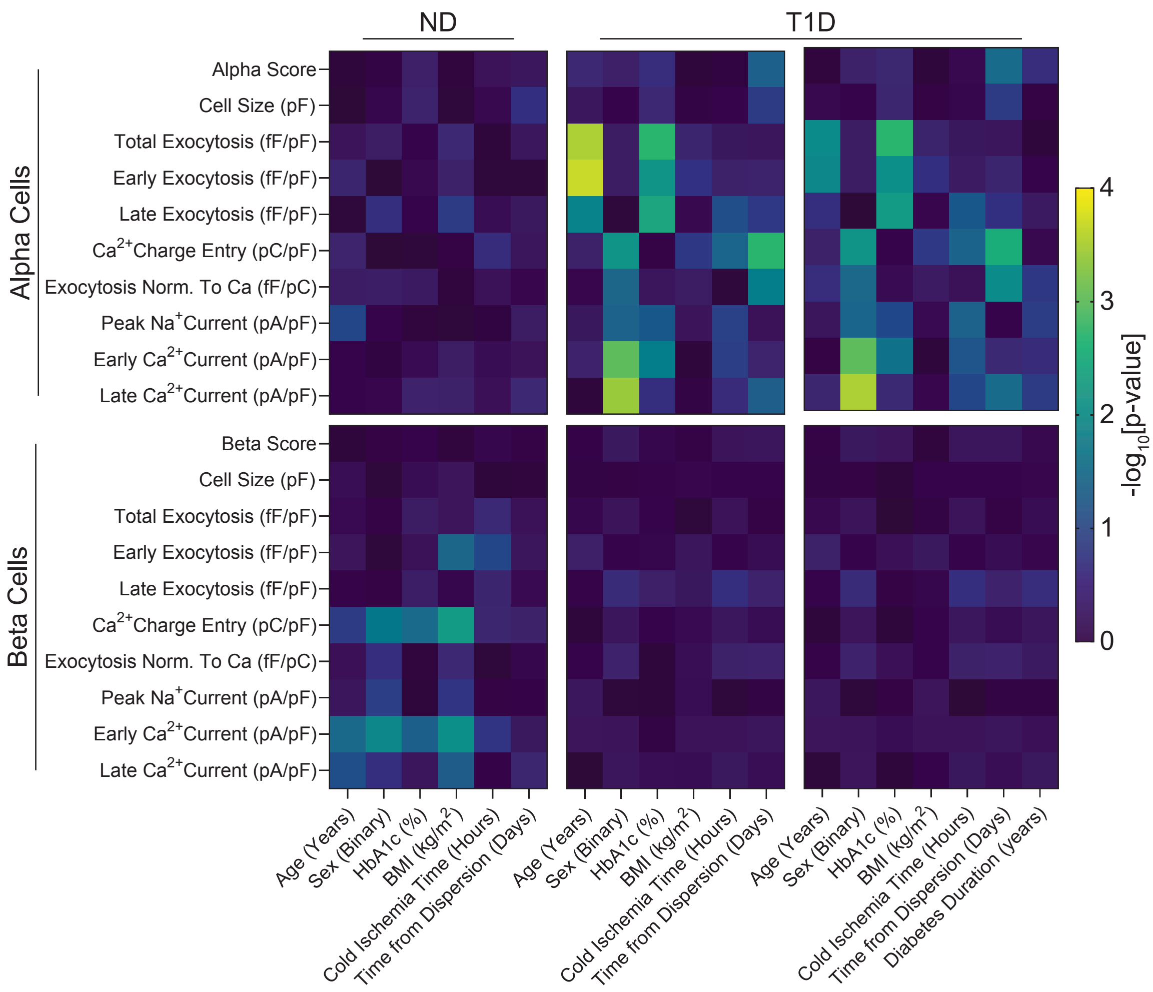

Supplemental Figure 7

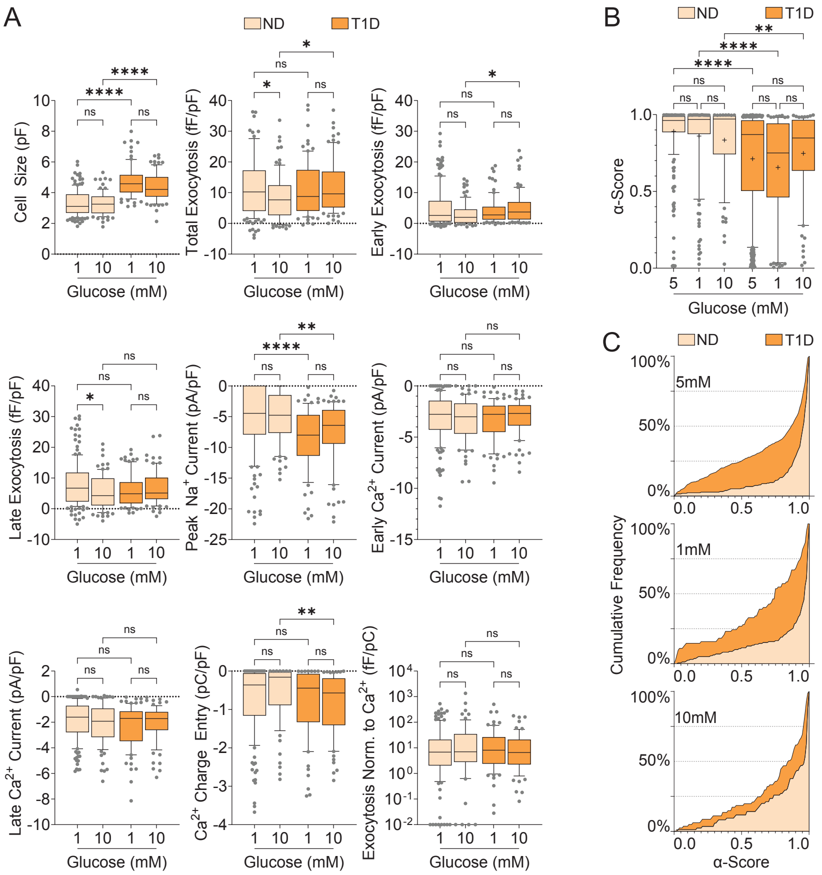

Supplemental Figure 8

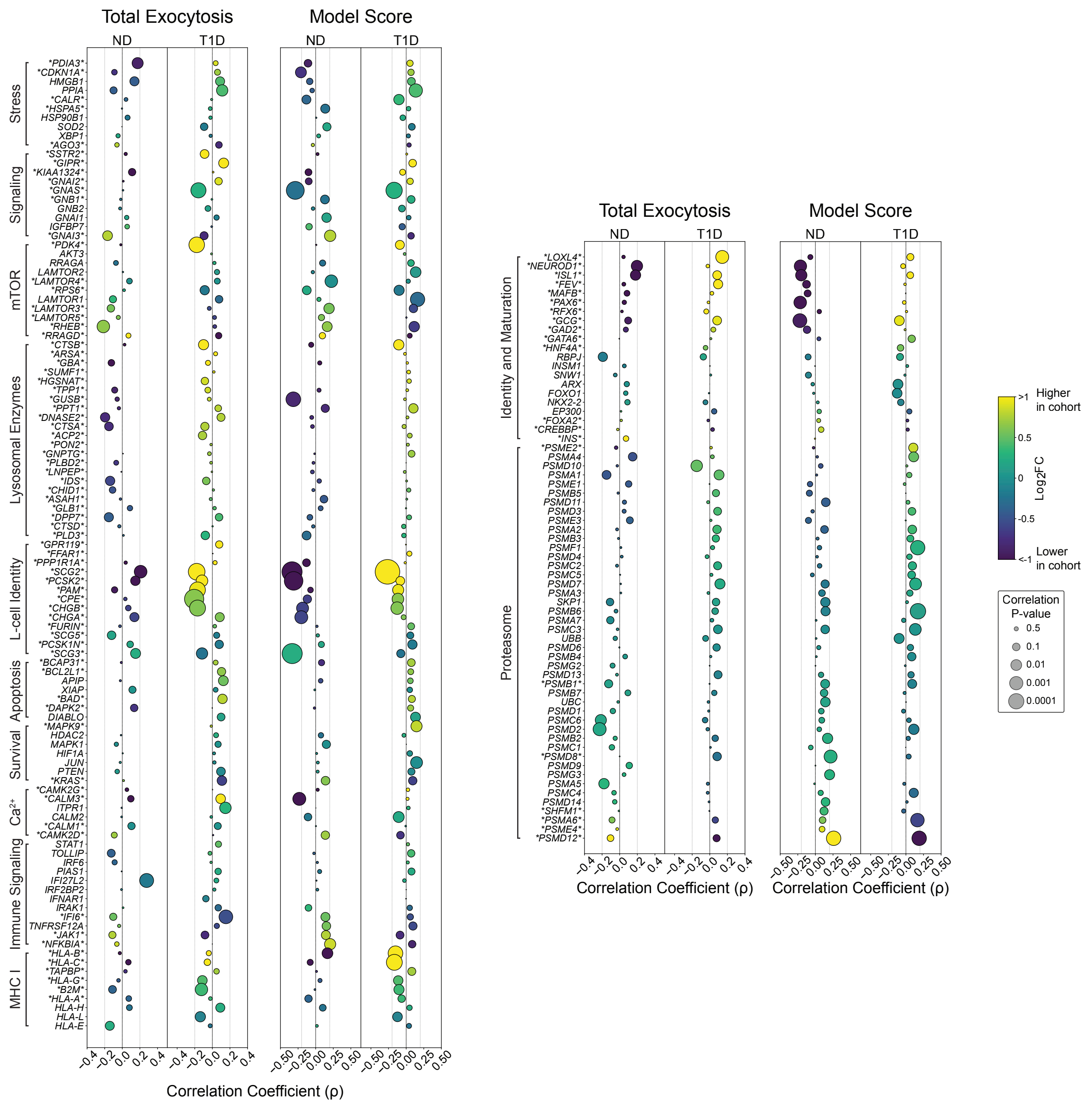

Supplemental Figure 9

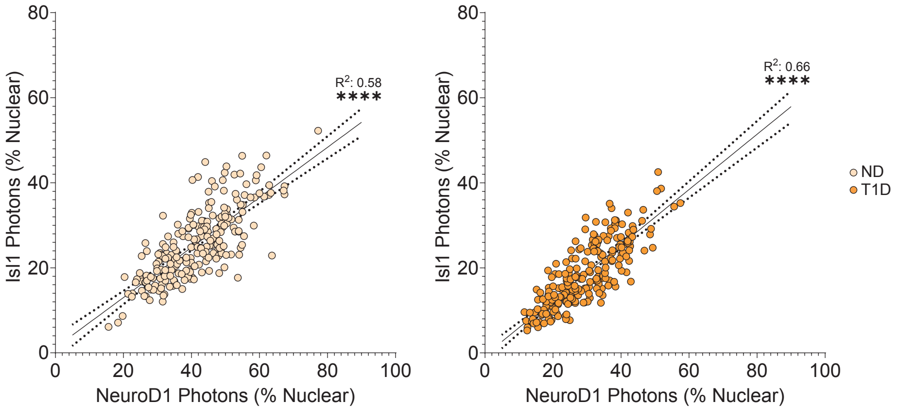

Supplement: Supplemental data [file jci-135-195267-s108.pdf]
